# Identification of critical residues in Hepatitis E virus macro domain involved in its interaction with viral methyltransferase and ORF3 proteins

**DOI:** 10.1038/srep25133

**Published:** 2016-04-26

**Authors:** Saumya Anang, Chandru Subramani, Vidya P. Nair, Sheetal Kaul, Nidhi Kaushik, Chandresh Sharma, Ashutosh Tiwari, CT Ranjith-Kumar, Milan Surjit

**Affiliations:** 1 Virology Laboratory, Vaccine and Infectious Disease Research Centre, Translational Health Science and Technology Institute, NCR Biotech Science Cluster, 3rd Milestone, Faridabad-Gurgaon Expressway, P O Box No. 04, Faridabad-121001, Haryana, India; 2 Centre for Bio-design & Diagnostics, Translational Health Science and Technology Institute, NCR Biotech Science Cluster, 3rd Milestone, Faridabad-Gurgaon Expressway, P O Box No. 04, Faridabad-121001, Haryana, India; 3 Experimental Medicine and Biotechnology Department, Postgraduate Institute of Medical Education and Research (PGIMER), Chandigarh, India

## Abstract

Hepatitis E virus (HEV) is a major cause of hepatitis in normal and organ transplant individuals. HEV open reading frame-1 encodes a polypeptide comprising of the viral nonstructural proteins as well as domains of unknown function such as the macro domain (X-domain), V, DUF3729 and Y. The macro domain proteins are ubiquitously present from prokaryotes to human and in many positive-strand RNA viruses, playing important roles in multiple cellular processes. Towards understanding the function of the HEV macro domain, we characterized its interaction partners among other HEV encoded proteins. Here, we report that the HEV X-domain directly interacts with the viral methyltransferase and the ORF3 proteins. ORF3 association with the X-domain was mediated through two independent motifs, located within its N-terminal 35aa (amino acids) and C-terminal 63-123aa. Methyltransferase interaction domain was mapped to N-terminal 30-90aa. The X-domain interacted with both ORF3 and methyltransferase through its C-terminal region, involving 66^th^,67^th^ isoleucine and 101^st^,102^nd^ leucine, conserved across HEV genotypes. Furthermore, ORF3 and methyltransferase competed with each other for associating with the X-domain. These findings provide molecular understanding of the interaction between the HEV macro domain, methyltransferase and ORF3, suggesting an important role of the macro domain in the life cycle of HEV.

Hepatitis E virus (HEV) causes acute viral hepatitis, which is a major public health concern in developing and resource poor countries^1^. The disease is mostly self-limiting but chronic infection has been reported in immune-compromised patients^2^,^3^. Moreover, HEV infection significantly increases the mortality rate (~30%) in pregnant women^4^. The virus is zoonotic and transmitted predominantly through the feco-oral route (contaminated food and water), blood and vertical transfusion^5^.

HEV is the only member classified as a *Hepevirus* in the family of *Hepeviridae.* It is a positive-sense, single strand, nonenveloped RNA virus with a 7.2 kb genome. It consists of a 5′-noncoding region (NCR) of 27 to 35 nucleotides, followed by three known open reading frames: ORF1, ORF2 and ORF3 and a 3′-NCR of 65 to 74 nucleotides, ending with a poly (A) tail of variable length^1^. The 5′ end has m7G cap^6^. The ORF1 encodes the nonstructural proteins: methyltransferase (Met), papain-like cysteine protease (PCP), helicase and RNA dependent RNA polymerase (RdRp). It also codes for domains of unknown functions such as X, Y, V and DUF3729 domain^7^. HEV methyltransferase is able to catalyze the transfer of methyl group from S-adenosyl methionine to GTP, to yield m7GTP and also forms a covalent complex with m7GMP, indicating an associated guanylyltransferase activity^8^,^9^. PCP has been reported to deubiquitinate interferon-stimulated gene-15 AMC (ubiquitin-7-amino-4-methylcoumarin)^8^. HEV helicase is a nucleoside triphosphatase with the ability to unwind RNA duplexes in the 5′ to 3′ direction^10^,^11^. Viral RdRp has been shown to be essential for viral replication^12^,^13^. ORF2 is an N-linked glycoprotein, which forms the viral capsid^14^. ORF2 glycosylation has been demonstrated to be important for the formation of infectious virus particles^15^. ORF2 binds to the 5′-region of HEV genomic RNA^16^. It has been shown to induce endoplasmic reticulum (ER) stress and exploit the ER-associated degradation pathway to gain access to the cytoplasm where it inhibits the host NFκB activity^17^,^18^. The ORF3 is a small phosphoprotein situated between ORF1 and ORF2, overlapping ORF2. It binds to several host proteins and modulates their activity^19^. Importantly, it binds to the tumor susceptibility gene101 (TSG101) and mediates the release of the progeny virus^20^,^21^.

Among the domains of unknown function, the X-domain is designated as a macro domain, owing to its homology with the macro domain of the histone H2A2^22^. Macro domains are conserved protein domains, widely distributed across bacteria, archaea and eukaryotes. Human genome contains nine genes encoding the macro domain proteins^23^: two histones macroH2A1 and macroH2A2 are involved in genome silencing and regulation of gene expression^24^; Snf2-like helicase (ALC1), a protein involved in chromatin remodeling; three proteins belonging to the B-aggressive lymphoma (BAL) family of transcription factors^25^,^26^; and the function of the other three, MDO1 (LRP16), MDO2 and MDO3 (GDAP2) is poorly understood.

The macro domains are also present in many positive strand RNA viruses such as the SARS CoV (Severe Acute Respiratory Syndrome CoV), the Sindbis virus and the Rubella virus, where it is referred as the X-domain^27^. The X-domain of the SARS-CoV and HEV has been shown to efficiently bind free and poly ADP–ribose polymerase-1 bound poly ADP-ribose *in vitro*. However, they have relatively poor ADP-ribose 1″-phosphohydrolase activity^27^. When overexpressed in HEK293T cells, the HEV X-domain inhibits poly (I:C) induced phosphorylation of the interferon regulatory factor 3 (IRF-3), which is the key transcription factor for interferon induction^28^. Mutational studies have indicated a role of the X-domain in viral replication^22^,^29^. However, the exact function of the X-domain in the life cycle of HEV remains to be explored.

This study was initiated to understand the role of the X-domain during the life cycle of HEV. We have identified the interaction partners of the X-domain among the HEV encoded proteins and demonstrate that the X-domain directly and selectively associates with the viral methyltransferase and the ORF3 proteins. Further investigations were carried out to map the interaction domain, evaluate the specificity and establish whether the interaction partners competed or cooperated with each other to form a multi protein complex. Significance of these interactions during the life cycle of HEV is discussed.

## Results

### HEV macro domain directly interacts with the viral methyltransferase and the ORF3 proteins

In order to identify the direct interaction partners of the HEV macro domain, a GAL4 transcription factor based Yeast Two Hybrid (Y2H) assay was designed. Two different host strains (*Y2H gold* and *Mav203*) were used to perform the assays. *Y2H gold* contains 4 independent reporters: *AUR1-C* [confers resistance to Aureobasidin A (A^r+^)], *HIS3* [permits growth on histidine deficient (H^−^) medium], *ADE2* [permits growth on Adenine deficient (A^−^) medium] and *MEL1* [codes for α-galactosidase, which produces blue color in the presence of X- α-gal]. These reporters are expressed upon successful interaction between the BD and the AD fusion proteins. Two different methods were followed to evaluate the interaction between the bait and the prey proteins in *Y2H Gol*d: (a) replica plating of the cotransformants into histidine deficient growth medium containing increasing quantities of 3-AT (3-amino 1,2,4-triazole), a competitive inhibitor of the enzyme imidazoleglycerol-phosphate dehydratase (encoded by the *HIS3* reporter gene), which catalyzes the sixth step in histidine biosynthesis pathway. If two proteins interact with high affinity, more imidazoleglycerol-phosphate dehydratase is produced, which requires higher quantity of 3-AT to be inhibited. Therefore, ability of the cotransformants to grow on increasing amount of 3-AT reflects the strength of interaction of the test proteins. Growth of the colonies were monitored after 3 days of replica plating and relative growth was scored in a scale of “+” to “+++” where +, ++ and +++ indicates poor, average and strong growth, respectively. (b) Quantitative estimation of α-galactosidase activity (encoded by the MEL1 reporter gene) by performing a liquid α-galactosidase assay *in vitro* in the presence of its substrate p-nitrophenyl-α-D-galactoside. *Mav203* contains *HIS3* [permits growth on H^−^ medium], *URA3* [permits growth on Uracil deficient (U^−^) medium] and *lacZ* [codes for β-galactosidase, which produce blue color in the presence of X-Gal] reporters to monitor the protein-protein interactions. Strength of the interaction between the bait and the prey proteins in *Mav203* was evaluated by scoring the colony growth on increasing concentrations of 3-AT, as mentioned above.

**Figure 1:**
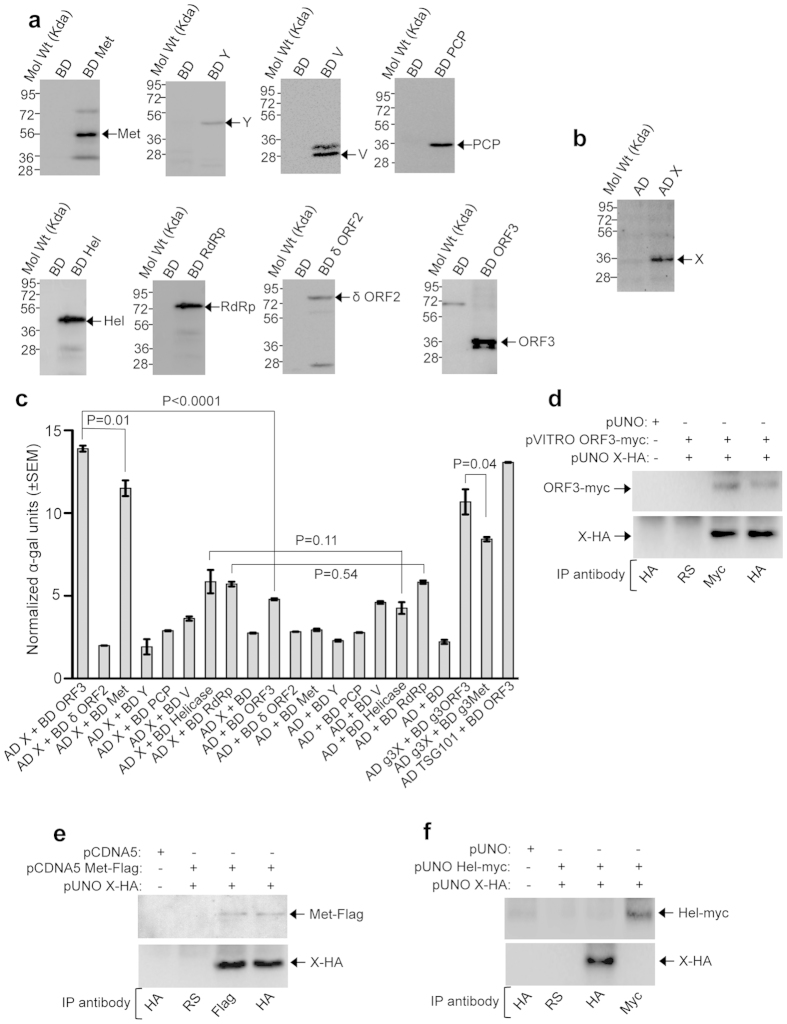
Association of the HEV X-domain with multiple viral proteins. (**a**) Western blot of *Y2H gold* whole cell extract expressing the indicated plasmids using anti-myc antibody to detect various viral proteins (as GAL4 binding domain fusion). BD: GAL4 binding domain expression plasmid. (**b**) Western blot of *Y2H gold* whole cell extract expressing the indicated plasmids using anti-HA antibody to detect the expression of GAL4 activation domain fused X-domain. AD: GAL4 activation domain expression plasmid. (**c**) Quantitative α galactosidase assay of Y2H gold cotransformants represented in Table 1. Normalized α galactosidase units are plotted as ± SEM of triplicate samples. (**d**) CoIP of ORF3 (ORF3-myc) and X-domain (X-HA) expressing Huh7 cell extract, immunoprecipitated using indicated antibodies and immunoblotted using anti-myc (upper panel) and anti-HA (lower panel) antibodies. RS: Rabbit pre immune serum. (**e**) CoIP of methyltransferase (Met-Flag) and X-domain (X-HA) expressing Huh7 cell extract, immunoprecipitated using indicated antibodies and immunoblotted using anti-Flag (upper panel) and anti-HA (lower panel) antibodies. RS: Rabbit pre immune serum. (**f**) CoIP of helicase (Hel-myc) and X-domain (X-HA) expressing Huh7 cell extract, immunoprecipitated using indicated antibodies and immunoblotted using anti-myc (upper panel) and anti-HA (lower panel) antibodies. RS: Rabbit pre immune serum.

All predicted domains of ORF1 polypeptide^7^ as well as ORF3, full length ORF2 and 112-608aa (amino acids) of ORF2 (δ ORF2) from a genotype-1 HEV isolate (SAR 55 strain, GI: AF444002) were cloned as C-terminal fusion of the GAL4 binding domain (BD) into the yeast expression vector pGBKT7. Note that in our laboratory, Y2H screening involving full length ORF2 did not identify any interaction partner, whereas 112-608aa of ORF2 could interact with a few host proteins (Subramani and Surjit, unpublished data). Therefore, in addition to the full length, 112-608aa of ORF2 was used in the current study. All constructs were transformed into *Y2H gold* along with an empty vector plasmid as negative control (BD) and expression of fusion proteins were confirmed by western blot of the whole cell extract of the transformed colonies using an antibody against the myc-tag, located between the GAL4 BD and our protein of interest. All the constructs produced expected size of the fusion protein (Fig. 1a). Similarly, the X-domain of HEV was cloned as a C-terminal fusion of the GAL4 activation domain (AD) into the yeast expression vector pGADT7 (named AD X) and its expression was confirmed by western blot of transformed *Y2H gold* whole cell extract using an antibody against the HA-tag, located between the GAL4 AD and the X-coding sequence (Fig. 1b).

AD X was transformed into *Y2H gold* along with the empty BD expression plasmid (pGBKT7) to monitor self-activation of the reporter genes. No background reporter activity was observed in the AD X+BD cotransformed colonies (Table 1 and Supplementary Fig. S1), ruling out the possibility of self-activation by the X-domain. A previously reported interaction between the BD ORF3 and AD TSG101^20^ was used as a positive control for the assay. ORF3 and TSG 101 cotransformants could grow well on LTHA^−^, LTH^−^A^r+^ and up to 20 mM 3-AT on LTH^−^ plates, indicating that the assay was working as expected (Table 1). Representative images of the colonies obtained from various cotransformants have been shown to correlate with the empirical scoring (Supplementary Fig. S1). In quantitative liquid α-galactosidase assay too, ORF3-TSG 101 cotransformed samples displayed significant α-galactosidase activity, compared to the vector controls (Fig. 1c). Next, AD X was transformed in pair with different HEV proteins followed by replica plating of the transformants onto different selection media. AD X, BD ORF3 and AD X, BD Met (methyltransferase) cotransformants could grow on the LTH^−^, LTHA^−^, LTH^−^A^r+^ plates and produced blue colonies on the LT^−^+X-α-gal plates, indicating that the X-domain interacted with both ORF3 and methyltransferase. Note that, neither ORF3 nor methyltransferase displayed self-activation, evident from the inability of AD+BD ORF3 and AD+BD Met cotransformants to activate any of the reporters (Table 1, Fig. 1c and Supplementary Fig. S1). Comparison of growth of the cotransformants on increasing concentrations of 3-AT supplemented LTH^−^ plates revealed that AD X+BD ORF3 could grow at 20 mM 3-AT (Table 1), whereas the AD X+BD Met could grow only up to 5 mM 3-AT (Table 1 and Supplementary Fig. S1). Further, the AD X+BD Met cotransformants also displayed poor growth on LTHA^−^, LTH^−^A^r+^ plates and less intense blue colonies on LT^−^+X-α-gal plates (Table 1 and Supplementary Fig. S1), suggesting a weak interaction of the X-domain with the methyltransferase, compared to that of the ORF3. Similar results were obtained in the quantitative α-galactosidase assay (Fig. 1c). The AD X and BD helicase cotransformants could activate only the *ADE2* and *HIS3* reporters, evident from their ability to grow on the LTHA^−^, LTH^−^ and LTH^−^+ 5 mM 3-AT plates. Neither did they grow on the LTH^−^A^r+^ plates nor displayed blue colonies on the LT^−^+X-α-gal plates (Table 1 and Supplementary Fig. S1). Furthermore, no significant difference was observed in the α-galactosidase activity between the AD X+BD helicase and AD+BD helicase cotransformants (Fig. 1c). In our hands, DUF3729 itself activated all reporters, rendering it unsuitable for the Y2H analysis. No other viral protein interacted with the X-domain. Similar results were obtained when the bait and prey domains were swapped (data not shown). The same experiment was performed using the *Mav203* yeast strain. In our laboratory conditions, both the *URA3* and *lacZ* reporters of *Mav203* displayed excessive background activity, ruling out their usefulness. Hence, cotransformants were grown on the LTH^−^ medium containing increasing concentrations of 3-AT to evaluate the interaction between the X and other viral proteins. In agreement with the *Y2H gold* data, X-domain interacted strongly with ORF3 than methyltransferase in the *Mav203* strain (Supplementary Table S1 and Supplementary Fig. S2). The X-domain and helicase cotransformants could grow up to 10 mM 3-AT on the LTH^−^ plates, as the negative controls (Supplementary Table S1 and Supplementary Fig. S2).

We also tested whether the interaction between X and ORF3 or methyltransferase was observed in the genotype-3 virus. Y2H analysis of the genotype-3 X fused to AD (AD g3X), genotype-3 ORF3 fused to BD (BD g3ORF3) and genotype-3 methyltransferase fused to BD (BD g3Met) produced similar results as in genotype-1 (Table 1, Fig. 1c, Supplementary Table S1, Figs S1 and S2). Collectively, these results indicate that the X-domain of both genotype-1 and genotype-3 HEV directly interacts with the ORF3 and the methyltransferase proteins.

A coimmunoprecipitation (CoIP) assay was performed to confirm the above observations. Huh7 human hepatoma cells were transiently transfected with expression plasmids coding for the C-terminal HA-tagged X (pUNO X-HA) and N-terminal myc-tagged ORF3 (pVITRO ORF3-myc) or empty vector (pUNO). 48 hours post-transfection, whole cell extract was immunoprecipitated (IP) with either rabbit pre immune serum (RS) or HA and myc antibodies. As expected, both HA and myc antibodies could pull down ORF3 and X-domain, respectively, confirming their association (Fig. 1d). Similarly, CoIP assay of Huh7 cells transiently expressing HA-tagged X and Flag-tagged methyltransferase (pCDNA5 met-Flag) confirmed the association between them (Fig. 1e). In contrast, the CoIP assay of Huh7 cells transiently expressing HA-tagged X and myc-tagged helicase (pUNO Hel-myc) did not reveal any association between them (Fig. 1f).

### Identification of the minimal region of interaction in the ORF3, the methyltransferase and the X-domain

**Figure 2:**
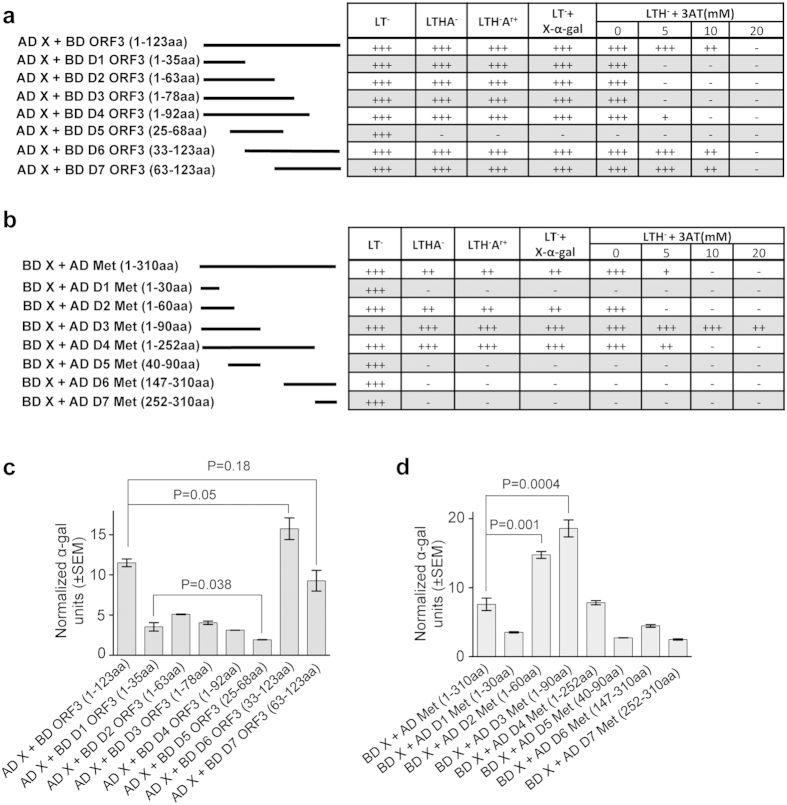
Mapping the region of interaction between X and methyltransferase or ORF3. (**a**) Yeast Two Hybrid (Y2H) analyses of the interaction potential of ORF3 deletion mutants with X-domain. Length of the deletion mutants is graphically represented as solid lines and amino acids of ORF3 retained in those deletions are mentioned in brackets. AD: Activation domain, BD: Binding domain, +++: Strong growth, ++: Moderate growth, +: Poor growth, −: No growth, L: Leucine, T: Tryptophan, H: Histidine, A: Adenine hemisulfate, A^r^: Aureobasidin, “−”: Deficiency in the medium, “+”: Supplemented in the medium. (**b**) Y2H analyses of the interaction potential of methyltransferase (Met) deletion mutants with the X-domain. Length of the deletion mutants is graphically represented as solid lines and amino acids of methyltransferase (Met) retained in those deletions are mentioned in brackets. AD: Activation domain, BD: Binding domain, +++: Strong growth, ++: Moderate growth, +: Poor growth, −: No growth, L: Leucine, T: Tryptophan, H: Histidine, A: Adenine hemisulfate, A^r^: Aureobasidin, “−”: Deficiency in the medium, “+”: Supplemented in the medium. (**c**) Quantitative α-galactosidase assay of *Y2H gold* cotransformants represented in Fig. 2a. Normalized α-galactosidase units are plotted as ± SEM of triplicate samples. (**d**) Quantitative α-galactosidase assay of *Y2H gold* transformants represented in Fig. 2b. Normalized α-galactosidase units are plotted as ± SEM of triplicate samples.

A series of deletion mutants of the ORF3, methyltransferase and X-domain were generated to map the minimal region of interaction. Y2H analysis of the interaction potential between the full length X-domain (AD X) and 7 overlapping deletion fragments of the ORF3 (BD D1-D7 ORF3, Fig. 2a) revealed that both the D6 (33-123aa) and the D7 (63-123aa) ORF3 deletions were equally efficient as the full length ORF3 for interaction with the X-domain (Fig. 2a). The D1-D4 ORF3 deletions weakly interacted with the X-domain. The D5 ORF3, which encompasses the N-terminal hydrophobic domain II, did not interact with the X-domain (Fig. 2a). Similar results were obtained in the quantitative liquid α-galactosidase assay (Fig. 2c). These results indicate that ORF3 associates with the X-domain through two independent domains, located at the N-terminus (within 1-35aa) and the C-terminus (within 63-123aa).

Y2H analysisof the interaction potential between the full length X-domain (AD X) and 7 overlapping deletions of the methyltransferase (BD D1-D7 met) revealed that removal of 210aa from the C-terminus significantly increased the strength of the interaction, (compare growth of full length, D3 met colonies on different media; Fig. 2b and α-galactosidase activity; Fig. 2d). N-terminal 1-60aa fragment (D2 met) also interacted with the X-domain (Fig. 2b,d). On the other hand, the D5-D7 met deletions (40-90aa, 147-310aa, 252-310aa) were unable to interact with the X-domain (Fig. 2b,d). The above data suggests that the minimal region of the methyltransferase protein required for its interaction with the X-domain is present within its N-terminal 90aa.

**Figure 3:**
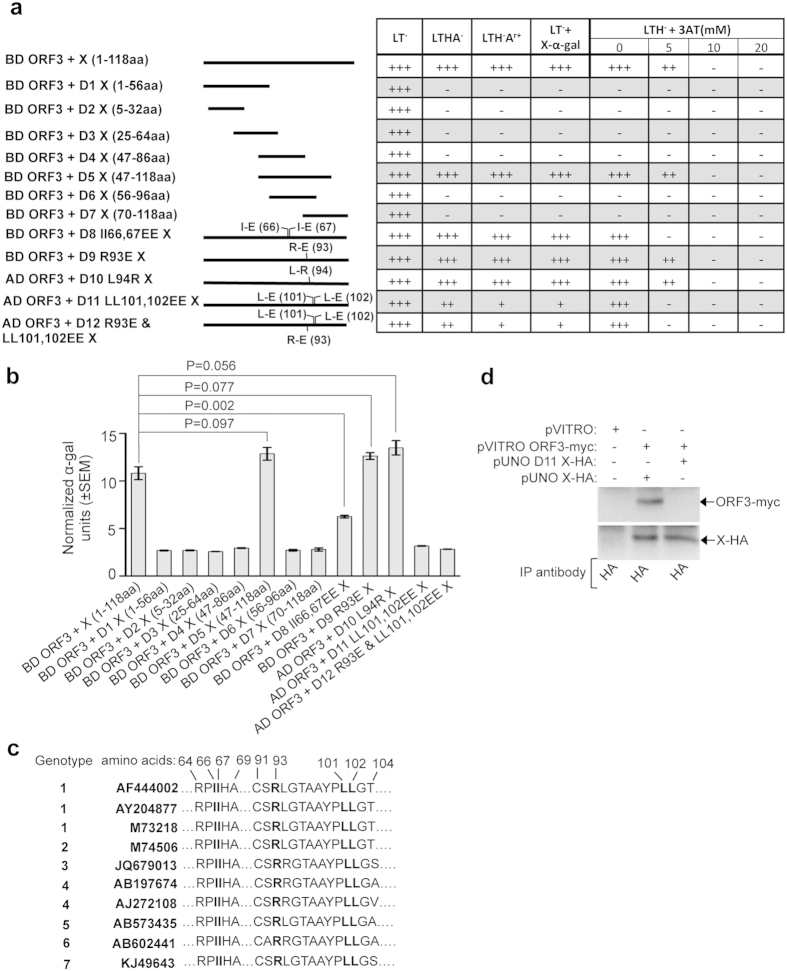
Deletion and point mutation analyses of the X-domain residues responsible for its interaction with the ORF3 protein. (**a**) Y2H analyses of the interaction potential of various deletion and point mutants of X with full length ORF3. Length of the deletion mutants is graphically represented as solid lines and amino acid position of point mutants are indicated in brackets. AD: Activation domain, BD: Binding domain, +++: Strong growth, ++: Moderate growth, +: Poor growth, −: No growth, L: Leucine, T: Tryptophan, H: Histidine, A: Adenine hemisulfate, Ar: Aureobasidin, “−”: Deficiency in the medium, “+”: Supplemented in the medium. (**b**) Quantitative α-galactosidase assay of *Y2H gold* transformants represented in Fig. 3a. Normalized α-galactosidase units are plotted as ± SEM of triplicate samples. (**c**) Alignment of X-domain sequence of different HEV isolates. Isoleucine 66^th^, 67^th^, Arginine 93^rd^ and Leucine 101^st^, 102^nd^ are indicated in bold letters. (**d**) CoIP of Huh7 cell extract expressing empty vector or wild type X (X-HA) and ORF3 (ORF3-myc) or LL-EE mutant X (D11 X-HA) and ORF3 (ORF3-myc), immunoprecipitated using anti-HA antibody and immunoblotted using anti-myc (upper panel) or anti-HA (lower panel) antibodies.

Next, a series of X-domain deletions were tested for their ability to associate with the ORF3 and the methyltransferase. 47-118aa (D5 X) of the X-domain was sufficient for interacting with the ORF3 (Fig. 3a). However, shorter fragments within the above region [56-96aa (D6 X) or 70-118aa (D7 X)] were unable to interact with the ORF3 protein (Fig. 3a). Secondary structure analysis of the X-domain from various HEV isolates using Mac Vector (Mac Vector Inc., North Carolina, USA) predicted the presence of two conserved β-sheet structures within the 47-118aa region, which were absent in the D6 X and D7 X mutants. Assuming that those structures were responsible for mediating the interaction between the X and ORF3, we planned to introduce point mutations at crucial amino acid positions within 47-118aa region to destroy the predicted β-sheets without deleting multiple amino acids at a stretch. Secondary structure analysis of the X-domain predicted that altering 66 and 67 isoleucines (I) to glutamic acid (E) II66,67EE lead to complete disappearance of the β sheet from 58^th^-70^th^aa. Altering 93 arginine (R) to glutamic acid (E) R93E or 94 leucine (L) to arginine (R) L94R lead to partial destruction of the β-sheet present between 90^th^-111^th^aa, whereas altering 101 and 102 leucines (L) to glutamic acid (E) LL101,102EE lead to almost complete disappearance of the same β-sheet. Except for the 94^th^ L, which was conserved in 60% sequences, all other altered amino acids (66^th^, 67^th^ I; 93^rd^ R and 101^st^, 102^nd^ L) were fully conserved across all HEV isolates analyzed (Fig. 3c). Secondary structure analysis of a triple mutant (R93E, LL101,102EE) predicted complete loss of the 90^th^-111^th^ β-sheet.

Above mentioned point mutations were introduced into the full length X-domain, followed by Y2H analyses. R93E or L94R substitutions were ineffective in preventing the interaction between the X and the ORF3 (Fig. 3a). However, both II66,67EE and LL101,102EE substitutions significantly impaired the ability of the X-domain to associate with ORF3, the latter being more effective (Fig. 3a). Triple mutation of R93E, LL101,102EE did not have any additive effect (Fig. 3a). Similar results were obtained in the quantitative α-galactosidase assay (Fig. 3b).

CoIP assays were performed to confirm the above observation. The LL101,102EE mutations were introduced into the pUNO X-HA plasmid (named pUNO D11 X-HA). Huh7 cells were transfected with the empty vector (pVITRO) or pVITRO ORF3-myc along with the wildtype X (pUNOX-HA) or LL-EE mutant X (pUNO D11 X-HA). Immunoprecipitation of the whole cell extract with HA antibody followed by immunoblotting with myc antibody demonstrated coimmunoprecipitation of ORF3 only from cells expressing the wild type X-domain, but not from cells expressing the LL101,102EE mutant X (Fig. 3d).

**Figure 4:**
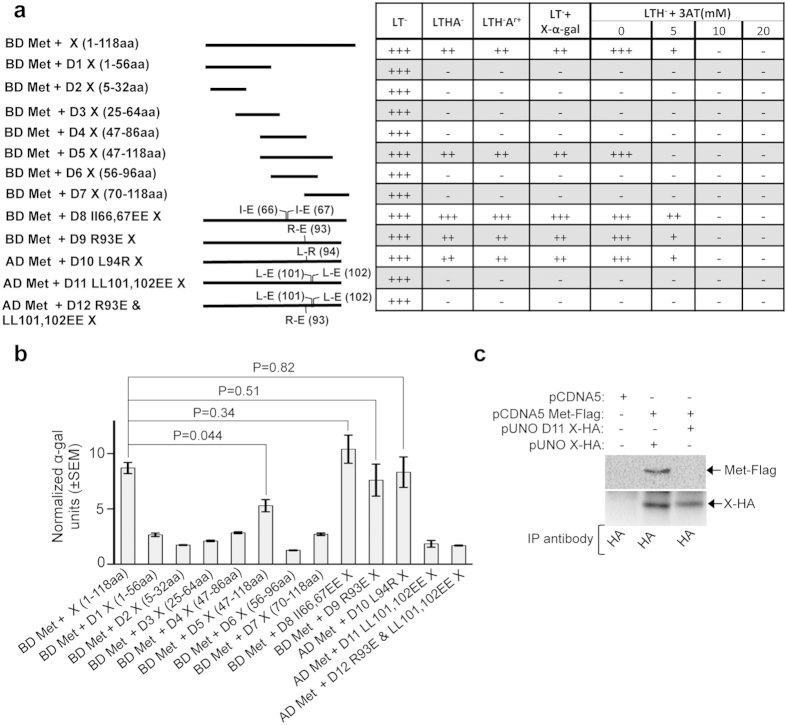
Deletion and point mutation analyses of the X-domain residues responsible for its interaction with the methyltransferase protein. (**a**) Y2H analyses of the interaction potential of various deletion and point mutants of X-domain with full-length methyltransferase (Met). Length of the deletion mutants is graphically represented as solid lines and amino acid position of point mutants are indicated in brackets. AD: Activation domain, BD: Binding domain, +++: Strong growth, ++: Moderate growth, +: Poor growth, −: No growth, L: Leucine, T: Tryptophan, H: Histidine, A: Adenine hemisulfate, Ar: Aureobasidin, “−”: Deficiency in the medium, “+”: Supplemented in the medium. (**b**) Quantitative α-galactosidase assay of *Y2H gold* transformants represented in Fig. 4a. Normalized α-galactosidase units are plotted as ± SEM of triplicate samples. (**c**) CoIP of Huh7 cell extract expressing empty vector or wild type X (X-HA) and methyltransferase (Met-Flag) or LL-EE mutant X (D11 X-HA) and methyltransferase (Met-Flag), immunoprecipitated using anti-HA antibody and immunoblotted using anti-Flag (upper panel) or anti-HA (lower panel) antibodies.

Y2H analyses of the ability of the X deletion mutants to associate with the methyltransferase displayed a pattern almost similar to that observed for the X and ORF3 interaction (Fig. 4a,b) except for the following differences. The II66,67EE substitution increased the strength of interaction with the methyltransferase, evident from increased growth on the LTHA^−^, LTH^−^A^r+^ and LTH^−^+5 mM 3-AT media (Fig. 4a). However, no significant increase in the α galactosidase activity was observed (Fig. 4b). Additionally, the LL101,102EE substitution completely abolished the ability of the X-domain to associate with the methyltransferase (Fig. 4a,b). Like ORF3, methyltransferase did not co-precipitate with the LL101,102EE mutant X (Fig. 4c).

**Figure 5:**
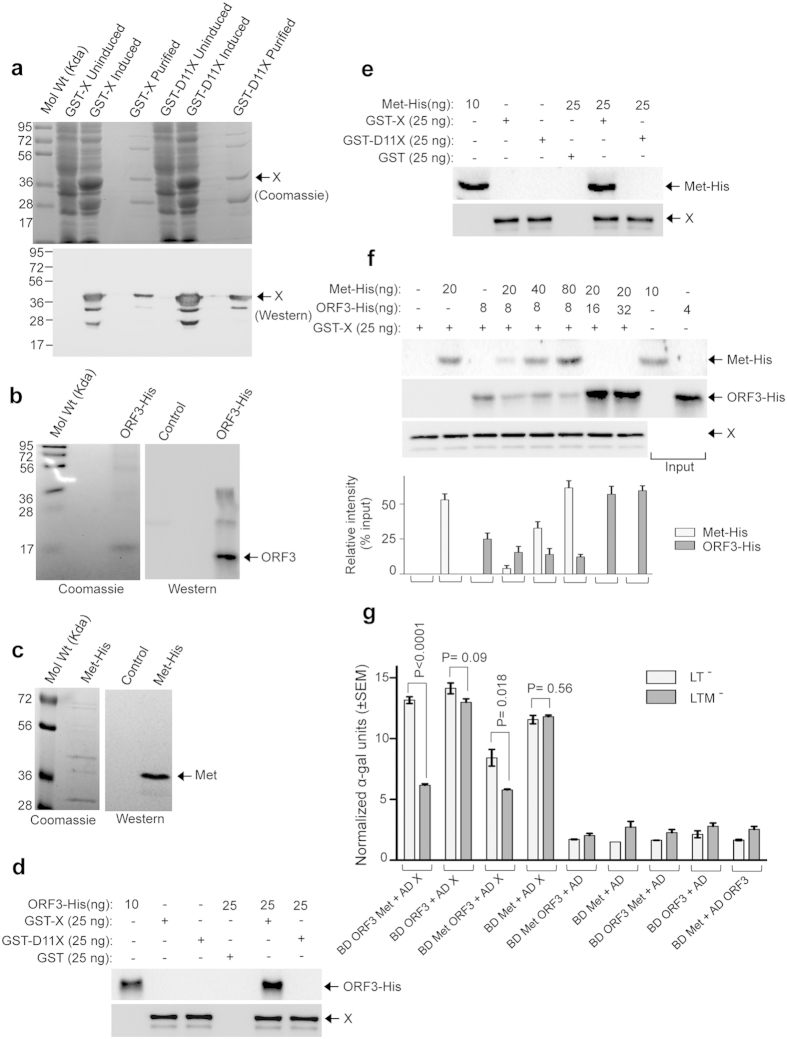
Competition between ORF3 and methyltransferase (Met) for binding to the X-domain. (**a**) Upper panel: Coomassie blue stained image of purified wildtype (GST-X) and LL-EE mutant (GST-D11) X-domain. Lower panel: Western blot image of upper panel samples, probed using anti-X antibody. (**b**) Left panel: Coomassie blue stained image of purified ORF3-His protein. Right panel: Western blot image of left panel sample, probed using anti-His antibody. (**c**) Left panel: Coomassie blue stained image of purified methyltransferase-His (Met-His) protein. Right panel: Western blot image of left panel sample, probed using anti-His antibody. (**d**) Pull down of His-tagged ORF3 protein using wild type (GST-X) or LL-EE mutant (GST-D11) X-domains as bait, revealed by immunoblotting using anti-His antibody (upper panel) and anti-X- antibody (lower panel). Only GST was used to control specificity of the binding (Lane 4). 40% of ORF3-His (10 ng) used in the pull down assay was loaded as input (Lane1). (**e**) Pull down of His-tagged methyltransferase using wild type (GST-X) or LL-EE mutant (GST-D11) X-domains as bait, revealed by immunoblotting using anti-His antibody (upper panel) and anti-X- antibody (lower panel). Only GST was used to control specificity of the binding (Lane 4). 40% of methyltransferase-His (10 ng) used in the pull down assay was loaded as input (Lane1). (**f**) Pull down of His-tagged ORF3 and His-tagged methyltransferase proteins using GST-X protein as bait, revealed by immunoblotting using anti-His-antibody (upper and middle panels) and anti-X antibody (lower panel). 50% of methyltransferase-His (10 ng) and ORF3-His (4 ng) used in the pull down assay was loaded as inputs in Lane 9 (upper panel) and lane 10 (middle panel), respectively. Relative band intensities of upper and middle panel images are plotted in the graph. (**g**) Quantitative α-galactosidase assay of *Y2H gold* transformants represented in Table 3. Normalized α-galactosidase units are plotted as ± SEM of triplicate samples.

Interactions between X, ORF3 and X, methyltransferase were further confirmed by performing a GST (Glutatione S-Transferase) pull down assay using partially purified recombinant proteins. GST fused X or LL101,102EE mutant X (D11 X) and His-tagged ORF3 or methyltransferase were affinity purified from *E. coli* using glutathione agarose and Ni-NTA superflow agarose resins, respectively. Expression, purification and specificity of the respective recombinant proteins were monitored by coomassie blue staining and western blot of the GST-X, GST-D11X and ORF3-His, methyltansferase-His proteins using anti-X and anti-His antibodies, respectively (Fig. 5a–c). Both ORF3 and methyltransferase could be pulled down by the wild type GST-X protein, but not by the GST-D11X protein (Fig. 5d,e). Specificity of the assay was ensured by incubating the ORF3 and methyltransferase proteins with GST, which did not produce any signal corresponding to ORF3 or methyltransferase (Fig. 5d,e).

### ORF3 and methyltransferase compete with each other for binding to the X-domain

Both ORF3 and methyltransferase lost the ability to interact with the LL101,102EE mutant X-domain, raising the possibility that they might compete with each other for binding to the X-domain. A pull down assay was performed to evaluate whether the ORF3 and methyltransferase competed with each other for binding to the X-domain. Approximately same molar equivalents (500 fmol) of the ORF3 and the methyltransferase proteins were allowed to bind with 500 fmol of the GST-X protein. Bound fractions were compared to that obtained by incubating 500 fmol ORF3 with GST-X or 500 fmol methyltransferase with GST-X. With reference to the input amount (50% loaded, Fig. 5f Lane 9, upper panel and Lane 10, middle panel), ~50% of methyltransferase and ~25% of ORF3 could be pulled down by the GST-X (Fig. 5f, Lane 2, upper panel and Lane 3, middle panel). Relative intensities of the bands have been quantified with reference to 100% of input protein and plotted in the accompanying graph (Fig. 5f, bottom panel). When all the three proteins were incubated together, ~5% of methyltransferase and ~10% of ORF3 could be detected in the bound fraction (Fig. 5f, Lane 4, upper and middle panel). Next, increasing quantities of either methyltransferase or ORF3 were used in the incubation mixture, keeping the quantity of ORF3 and GST-X or methyltransferase and GST-X constant. Increasing the methyltransferase amount by two and four fold molar excess increased its level in the bound fraction, simultaneously decreasing the level of ORF3 in the same fraction (Fig. 5f, Lanes 5 and 6, upper and middle panels). However, increasing the ORF3 quantity by two fold molar excess completely abolished the methyltransferase interaction (Fig. 5f, Lane 7), indicating that ORF3 possesses higher affinity for association with the X-domain compared to that of methyltransferase. These data also demonstrate that no cooperativity exists between the ORF3 and the methyltransferase for binding to the X-domain.

A Yeast Three Hybrid (Y3H) assay^30^ was also performed to confirm the above observations in an *in vivo* set up. ORF3 was fused to the BD in MCS1 (Multiple Cloning Site 1) of pBRIDGE vector and methyltransferase was cloned in the MCS2, under control of the MET 25 promoter (active only in the absence of methionine). The BD ORF3-Met and AD-X cotransformants revealed that ORF3 interacted with the X-domain in the presence of methionine (1mM methionine was supplemented in the medium to suppress MET25 promoter activity). Upon replica plating of the same colonies onto various selection media lacking methionine, growth was observed only on the LTM^−^ plates (Table 2; see also Supplementary Fig. S3). No growth was observed on LTHAM^−^, LTHM^−^A^r+^, LTHM^−^+5mM 3-AT, LTHM^−^+10mM 3-AT plates; neither were there blue colonies on the LTM^−^+X-α-gal plate (Table 2; see also Supplementary Fig. S3) indicating that the methyltransferase interfered with the interaction between ORF3 and the X-domain. Similar results were obtained in the quantitative α galactosidase assay (Fig. 5g). As a control to rule out a possible toxic effect of lack of methionine on the observed phenotype, ORF3 and X-domain interaction was monitored, which was not affected by methionine deficiency (Table 2, row 2; Fig. 5g; see also Supplementary Fig. S3). Similar result was obtained when methyltransferase was fused to BD, X-domain was fused to AD and ORF3 was inducibly expressed under control of the MET 25 promoter (Table 2, rows 3 and 4; Fig. 5g; see also Supplementary Fig. S3). We also observed that ORF3 and methyltransferase did not directly interact with each other (Table 2, row 9; Fig. 5g; see also Supplementary Fig. S3) thus ruling out the possibility that both of them may reside as a complex, away from the X-domain.

Taken together, both the pull down and Y3H assays demonstrate that ORF3 and methyltransferase compete with each other for binding to the X-domain and they do not form a trimeric complex. Further, as there is no direct interaction between ORF3 and methyltransferase, relative abundance and affinity of these proteins dictate their ability to associate with the X-domain.

### Specificity of the interaction between the HEV macro domain and RNA methyltransferase

Macro domains and RNA methyltransferase are present in a wide range of living organisms, including human, which is a host for the HEV. Since the HEV macro domain and RNA methyltransferase interacted with each other, we wondered whether such interactions are also prevalent in human and whether the virus and the host counterparts cross-talk with each-other.

Y2H analysis of the HEV X-domain interaction with 4 different human RNA methyltransferases did not provide any positive result (Table 3a). Y2H analysis of the HEV methyltransferase interaction with 7 human macro domain proteins revealed its weak interaction with the C20orf133 (Table 3b). Note that BD Met + AD LRP16 interaction was not considered significant as it activated only two reporters out of four (Table 3b).

Next, Y2H analysis was conducted to identify possible interactions between human macro domain proteins and RNA methyltransferases. Out of the 4 human RNA methyltransferases and 7 macro domain proteins tested, only human CMTR2 was able to associate with C20orf133 (Table 3c). These experiments indicate that the interaction between the macro domain proteins and the RNA methyltransferases might be important for optimal activity of either or both the proteins because such interactions are maintained not only in the HEV but also in higher eukaryotes like human.

## Discussion

In this study, we have identified and characterized the intra-viral interaction partners of the X-domain, which is a macro domain protein encoded by the HEV. Three independent assays were conducted to validate the true interaction partners: (a) Y2H assay identified direct interaction partners of the X-domain in a heterologous and *in vivo* set-up. Use of two different yeast strains and domain swapping ruled out the possibility of experimental artifacts; (b) CoIP assays established that the interactions obtained in Y2H assay were strong enough to be reproduced in human hepatoma cells, in the presence of many other potential host interaction partners of the X, methyltransferase and the ORF3; (c) an *in vitro* pull down assay using partially purified recombinant proteins confirmed that the two interaction partners possess the required motif/signature sequence to associate with each other. Only those viral factors, which could interact with the X-domain in the above three assays were considered as true interaction partners. Therefore, according to our data, only viral methyltransferase and ORF3 happen to be the direct intra-viral interaction partners of the X-domain. Viral helicase activated only two of the four reporter genes in the *Y2H gold* based assay. Neither did it show positive interaction in the *Mav203* based Y2H assay nor associated with the X-domain in the CoIP assay. Therefore, it is not a direct interaction partner of the X-domain. This finding is in contrast to a recent report published by Osterman and coworkers^31^, who reported helicase to associate with the X-domain. However, it is noteworthy that in both their assays, bait and prey were fusion proteins, (fused to BD and AD in Y2H assay and MBP and eGFPLuc in LuMPIS assay), which might have influenced their interaction properties. In our CoIP assay, X and helicase proteins contained only 8 extra amino acids (contain HA and Myc tags, respectively). Therefore, these proteins are more likely to behave like those produced during the natural course of infection.

Our efforts at mapping the region within the ORF3 protein that interacts with the X-domain identified two distinct regions: an N-terminal hydrophobic domain I and a C-terminal proline rich region^1^. Several studies have mapped ORF3 interaction with other viral and host proteins. Notably, ORF3 is known to self-associate and interact with SH3 domain containing proteins and bikunin through the C-terminal 43aa^32^,^33^,^34^. It also interacts with ORF2 through the proline rich region involving 57-81aa^35^, with hemopexin through the N-terminal hydrophobic domain II^36^ and with the Bβ fibrinogen through 63-123aa region^37^. Ability of ORF3 to interact with the X-domain through two independent binding domains may be an important strategy to promote or limit the binding of other factors to the same region depending upon the need of the virus.

Viral methyltransferase interacted with the X-domain through its N-terminal region, the N-terminal 30-60aa being the minimum sequence required for the association. N-terminal 1-60 and 1-90aa of the methyltransferase could grow better than the full length protein and displayed more α galactosidase activity, indicating their stronger binding with the X-domain than the full length methyltransferase. These data also suggests that the C-terminal region might be imposing structural constraints by masking the motif(s) required for associating with the X-domain or some other host protein may bind to the C-terminal region in such a manner that the N-terminal motif(s) remain inaccessible. Since HEV methyltransferase is known to be involved in capping of the 5′-end of the viral genomic RNA^6^,^9^, it would be interesting to investigate whether the X-domain controls capping of the viral genome by modulating the activity of methyltransferase. In this context, it is worth mentioning that the X-domain binding region identified in our study (30-90aa) lies almost inside the putative core methyltransferase domain of the HEV, which spans from 22-112aa of the methyltransferase protein used in our study (corresponding to 56-146aa of HEV ORF1). This domain is conserved among the Sindbis-like viruses such as Rubella, Semliki forest and Ross River virus^38^.

Our study also reveals that the interaction between RNA methyltransferase and macro domain proteins is not restricted to the HEV. In our limited analyses, we observed that one of the human RNA methyltransferases, CMTR2, interacts with the human macro domain protein C20orf133. CMTR2 is a cap specific RNA methyltransferase having mRNA (nucleotide-2′-O ribose) methyltransferase activity. It is involved in 7-methylguanosine mRNA capping, like the HEV methyltransferase and is also conserved in a broad range of organisms^39^. C20orf133/MACROD2 is a macro domain containing protein. Though its exact function remains to be established, it has been shown to be disrupted in patients with Kabuki syndrome^40^. Further studies are required to understand the functional significance of the interaction between CMTR2 and C20orf133, if any. Nevertheless, it is tempting to speculate that the interaction could be crucial for CMTR2 activity. Inspired by its host, the HEV might have evolved a similar strategy to maintain optimal methyltransferase activity.

Deletion and subsequent point mutation studies identified that two consecutive leucine residues (101, 102) located towards C-terminus of the X-domain are essential for its binding with both ORF3 and methyltransferase. Comparison with known protein binding motifs did not indicate any homology of the dileucine-containing region to known motifs. Thus, it is unlikely that the two leucine residues are essential amino acids of a linear motif. It could be possible that they maintain a favourable structure or permissive electrostatic environment for binding of the X-domain with the ORF3 or methyltransferase. Changing leucine to glutamic acid might be collapsing the structure or altering the electrostatic environment (note that leucine contains an aliphatic side group whereas glutamic acid contains a negatively charged side group). Similar mechanism may hold true for the observed effect of the II-EE mutation.

Our data also suggests that the methyltransferase differs from the ORF3 in binding to the X-domain because the II66,67EE mutation, which partly reduced the binding of the ORF3 to the X-domain, had no effect or mild stimulatory effect on the methyltransferase binding to the X-domain. Thus, the methyltransferase and the ORF3 might be associating with different epitopes/conformations/structurally distinct regions of the X-domain. Structural studies are essential to delineate the actual mechanism. Nevertheless, genetic mutants identified in this study will be instrumental in investigating the functional significance of the interaction between X and methyltransferase or ORF3 during the life cycle of HEV. The II66,67EE and LL101,102EE mutations can be introduced in the HEV genome to investigate the functional significance of the interaction between the ORF3, methyltransferase and the X-domain. It is worth mentioning that the C-terminal region of the HEV macro domain is highly conserved: the 66, 67 isoleucine and the 101, 102 leucines are absolutely conserved across all genotypes and isolates of the HEV. Moreover, 101, 102 leucines are also conserved in the macro domains of other positive strand RNA viruses such as Rubella, Sindbis, SARS and Semliki forest Virus. It would be interesting to investigate whether the macro domains of these viruses interact with their methyltransferases.

Through Y3H and GST-pull down assay, we have demonstrated that the ORF3 protein displays a higher affinity for binding with the X-domain than the methyltransferase. ORF3 is a viral late protein, synthesized from the subgenomic RNA, which is generated after replication of the genomic RNA^1^. ORF3 has been shown to be dispensable for viral replication but essential for release of the progeny virus from infected cells^41^,^42^,^43^. On the other hand, methyltransferase, which is involved in capping of the viral genome, seems to be important during early stages of the viral life cycle. Assuming that the X-domain is involved in both replication and release of the progeny virus, it might be worth speculating that the X-domain associates with and modulates the activity of the viral methyltransferase during early stages of the infection. At a later stage, ORF3 sequesters away the X-domain from associating with the methyltransferase by virtue of its ability to associate with the X-domain with a higher affinity. This shifts the focus of the virus from replication to packaging and release of the progeny. Future studies are directed at exploring the validity of the above hypothesis and revealing the functional significance of the interaction between the viral macro domain, methyltransferase and ORF3 proteins. If true, specific inhibitors that block the interaction of the X-domain with methyltransferase and ORF3 might prove to be an effective therapeutic against HEV.

## Methods

### Chemicals and reagents

Yeast vectors, *Y2H gold* strain, Aureobasidin A (630466) and X-α gal (630463) were from Clontech (Mountain View, California, USA). *Mav203* strain was from Life Technologies (Carlsbad, California, USA). Amino acid supplements, 3-Amino-1, 2, 4-triazole (3-AT, A8056), IPTG (I6758) were from Sigma-Aldrich (Saint Louis, Missouri, USA). Anti-His (SC-57598), anti-myc (SC-789) and anti-Flag (SC-807) antibodies were from Santa Cruz Biotechnology (Dallas, Texas, USA). Lysozyme (10153516103) was from Roche (Indianapolis, Indiana, USA). Ni NTA agarose (25215) and Glutathione agarose (16100) were from Thermo Scientific (Waltham, Massachusetts, USA). Peptide affinity purified rabbit polyclonal antibody against the macro domain (X) of HEV was generated at Genscript (Piscataway Township, New Jersey, USA) using KLH (Keyhole Limpet hemocyanin) conjugated peptide antigens. Peptide sequence is: CPDYRLEHNPKRLEA. Pre immune serum was collected from the animals prior to immunization. ELISA using peptides as antigen and pre immune serum as negative controls confirmed their titres to be >1:512,000. In competition assays, corresponding peptides could block the binding of cognate antibodies. Functionality of the antibody in western was validated by testing its ability to detect the purified X-domain (from bacteria) and the X-domain expressed in Huh 7 cells.

### Cloning

Desired fragments were either PCR amplified using specific primers and template or isolated by restriction digestion and ligated into the target vector following standard protocols^44^. Details of the procedure for individual clones are listed in Supplementary Table S2 and primer sequences are listed in Supplementary Table S3. All clones were verified by restriction mapping and confirmed by sequencing.

### Yeast Transformation and whole cell extract preparation

*Y2H Gold* and *Mav203 strains* were transformed using Lithium Acetate, following manufacturer’s instruction. Transformation mixture was plated on LT^−^ Synthetic Dropout media. Three days post-transfection, colonies were used for replica plating, whole cell extract preparation or quantitative liquid α-galactosidase assay. For whole cell extract preparation, spheroplasts were prepared by treatment with Zymolyase (5units/10^6^ cells), followed by lysis in Laemmli buffer (62.5 mM Tris-Cl pH 6.8, 2% SDS, 10% glycerol, 50mM DTT, 0.01% bromophenol blue).

### Yeast Two Hybrid assay

Lithium Acetate transformed *Y2H Gold* or *Mav203* cells were grown on LT^−^ plates, followed by replica plating into required selection medium. For *Y2H Gold*, LTH^−^, LTHA^−^, LTH^−^A^r+^, LT^−^ +X-α gal, LTH^−^+5 mM 3-AT, LTH^−^+10 mM 3-AT and LTH^−^+20 mM 3-AT were used. Plates were grown for 3 days at 30 °C in a humidified incubator, followed by visual scoring of colony growth in a scale of “+” to “+++” where +, ++ and+++ indicates poor, average and strong growth, respectively.

### Yeast Three Hybrid assay

pBRIDGE vector containing GAL4-BD fused ORF3 cloned under the control of constitutive ADH1 promoter (in MCS1) and methyltransferase in a second expression cassette (in MCS2), under the control of methionine deprivation induced MET25 promoter, was used to perform the Yeast Three Hybrid assay^30^. A second construct was also generated in which methyltransferase was inserted in the MCS1 and ORF3 in the MCS2. These vectors were cotransformed into *Y2H gold* along with the AD X, in the required combination and plated on LT^−^ plates supplemented with 1 mM methionine (compared to 0.13 mM methionine in regular Y2H selection plates). Eight random colonies from each plate were replica plated into LTHA^−^, LTH^−^ A^r+^, LT^−^+X-α-gal, LTH^−^+5 mM 3-AT and LTH^−^+10 mM 3-AT plates, supplemented with 1 mM methionine. A second set of replica plating was done into LTM^−^ plates to induce MET 25 promoter dependent protein expression from MCS2. Four days post incubation at 30 °C in a humidified chamber, colonies from LT^−^ or LTM^−^ plates were again replica plated into various selection media containing 1 mM methionine or no methionine. Colony growth was scored on day four-post incubation. Data are representative of three independent experiments.

### Quantitative liquid α-galactosidase assay

A liquid α-galactosidase assay was performed to quantitate the α-galactosidase activity in *Y2H Gold*, following manufacturer’s instruction (Clontech, Mountain View, CA, USA). Briefly, *Y2H Gold* cotransformants expressing the test protein pairs to be analyzed were inoculated in 5 ml of LT^−^ or LTM^−^ medium in triplicates and grown till the A_600_ reached ~1.0. A_600_ of each culture was noted for downstream calculations. Cells were collected by centrifugation at 3000 g, resuspended in 100 μl of Z buffer (40 mM Na2HPO4.7H2O, 60 mM NaH2PO4, 10 mM KCl, 1 mM MgSO4.7H2O, 50 Mm βME, pH 7.0), lysed by three times freeze-thaw in liquid nitrogen, followed by centrifugation at 10,000 g for 10 minutes to clarify the extract. 16 μl of the extract was assayed in triplicate for each sample in a 96 well plate. 43 μl of assay buffer (340 mM sodium acetate, pH 4.5, 33.3 mM p-nitrophenyl-α-D-galactoside) was added to each well and incubated at 30 °C for 60 minutes. Reaction was terminated using 136 μl of stop solution (1 M Na2CO3 in deionized H2O). Absorbance was measured at 410 nm. α-galactosidase units were calculated using the following formula after subtracting the obtained readings from that of the blank sample. [milliunits/(ml × cell)] = A_410_ × V_f_ × 1,000/[(ε × b) × t × V_i_ × A_600_], t = elapsed time (in min) of incubation, V_f_ = final volume of assay, V_i_ = volume of culture medium supernatant added, A_600_ = Absorbance of overnight culture, ε × b = p-nitrophenol molar absorptivity at 410 nm × the light path (cm) = 10.5 (ml/μmol) for 200-μl. α-galactosidase units are represented as mean ± SEM, analyzed using “GraphPad Prism 5.0” by the student t- test (two-tailed). P < 0.05 was considered significant.

### Mammalian cell culture and Transfection

Huh7 human hepatoma cells were maintained in Dulbecco’s modified Eagle medium (DMEM) containing 10% Fetal Calf Serum (FCS), 50 I.U./mL Penicillin and Streptomycin, in 5% CO_2_. Cells were transfected using Lipofectamine 2000, following manufacturer’s protocol (Life Technologies, Carlsbad, California, USA).

### Coimmunoprecipitation and Western Blotting

48 hours post transfection; cells were washed once with phosphate-buffered saline (PBS: 10 mM PO_4_^3−^, 137 mM NaCl, 2.7 mM KCl) and lysed in 500 μl of Co-immunoprecipitation (CoIP) buffer (20 mM Tris-Cl, pH 7.5, 150 mM NaCl, 1% Triton X-100, 1 mM EDTA, 1 mM EGTA, 2.5 mM sodium pyrophosphate, 1 mM β-glycerol phosphate, 1 mM Na_3_VO_4_) supplemented with protease inhibitor cocktail (Roche, Indianapolis, Indiana, USA). The lysates were clarified by centrifugation for 10′ at 13,000 g. For co-immunoprecipitation, an equal amount of protein was incubated with 1 μg of corresponding antibody overnight at 4 °C, followed by incubation with 100 μl of 10% protein A-sepharose beads for 1 hour. The beads were washed three times in the CoIP buffer, incubated at 95 °C in Laemmli buffer (62.5 mM Tris-Cl pH 6.8, 2% SDS, 10% glycerol, 50 mM DTT, 0.01% bromophenol blue) for 5′ and resolved by SDS-PAGE, followed by western blotting using required primary antibodies and corresponding horseradish peroxidase-conjugated secondary antibodies. Protein signals were detected by Enhanced Chemiluminiscent using a commercially available kit (Biorad, Hercules, California, USA).

### Protein Purification

Wild type and LL-EE mutant X was cloned as GST fusion into bacterial expression vector pGEX4T-1 and transformed into BL-21pLysS cells. Cells were grown to A_600_ ~0.5 and induced with 1 mM IPTG for 16 hours at 18 °C. Pellet was resuspended in Buffer A (PBS, 1% Triton X 100, protease inhibitor cocktail) and treated with 1 μg/ml lysozyme followed by sonication. Soluble proteins were incubated for 30 minutes with Glutathione agarose beads, followed by washing thrice in PBS. The fusion protein was eluted by incubation in 20 mM Glutathione in 50 mM Tris (pH- 8.0). N-terminal His-tagged methyltransferase cloned in pET28a vector was transformed into Rosetta pLysS cells. Cells were grown to A_600_ ~0.5 and induced with 0.5 mM IPTG for 16 hours at 18 °C. Pellet was resuspended in Buffer B (100 mM Sodium Phosphate buffer + 500 mM NaCl, pH-7.0, supplemented with protease inhibitor cocktail) and treated with 1 ug/ml of lysozyme followed by sonication. Soluble methyltransferase protein was precipitated in 30% Ammonium Sulphate, resuspended in buffer C (Buffer B + 20 mM imidazole) and incubated with Ni-NTA superflow agarose for 2 hours at 4 °C, followed by washing thrice in Buffer C. Bound protein was eluted in 500 mM imidazole. His-tagged ORF3 expression vector (pRSET ORF3) has been described^45^. pRSET ORF3 was transformed into BL21 (DE3) cells. Transformants were grown to A_600_ ~0.5, induced with 0.5 mM IPTG for 16 hours at 37 °C, resuspended in Buffer D (50 mM Tris, pH-8.0, 250 mM NaCl, 1 mM PMSF) and sonicated. Inclusion bodies were recovered, solubilized in Buffer E (50 mM Tris, pH-12.5, 250 mM NaCl, 20 mM β-mercaptoethanol, 3 M Urea) and incubated at room temperature for 1 hour, followed by centrifugation at 15,000 g for 20′ at 10 °C. Supernatant was diluted in Buffer F (50 mM Tris, pH-8.0, 250 mM NaCl, 10% glycerol) and loaded on HisTrap FF column (GE Healthcare, Little Chalfont, UK). Bound protein was eluted using Buffer G (Buffer F + 250 mM imidazole). Imidazole was removed by dialysis. In all cases, purification steps were monitored by SDS-PAGE followed by Coomassie blue staining and western blotting using anti-X (for GST-X), anti-His (for methyltransferase and ORF3) and anti-ORF3 (for ORF3) antibodies. Approximate concentrations of purified proteins were determined by running them along with known quantities of purified Bovine Serum Albumin and quantifying the band intensities.

### Pull down assays

A pull down assay was conducted using the purified wild type X (GST-X) or LL-EE mutant (GST-D11) X proteins as bait. 25 ng of the GST-tagged protein was mixed with the same quantity of purified ORF3-His or Met-His in PBS containing protease inhibitor cocktail, 25 μl of Glutathione agarose beads and incubated on a flip flop shaker at 4 °C for 1 hour. Samples were centrifuged at 500 g, 1′, 4 °C to settle the beads containing the bound proteins. Beads were washed thrice in PBS + 0.1% Triton X 100. Bound proteins were eluted by incubation at 95 °C for 5′ in Laemmli buffer. Aliquots of the eluted proteins were resolved by SDS-PAGE and western blotted using anti-X and anti-his antibodies. 40% (10 ng) of proteins used for the pull down assay were resolved as input.

For monitoring competition between the methyltransferase and the ORF3 proteins for binding to the X-domain, a pull down assay was conducted by using approximately same molar equivalents of each protein (~500 fmol) in the binding mixture. Based on the size of the fusion protein and quantitation obtained from comparison with BSA standard in coomassie stained gel, 1 pmol of GST-X, Met-His and ORF3-His corresponded to 50 ng, 40 ng and 16 ng proteins, respectively. For comparing the affinity of interaction, increasing quantity of the Met-His or ORF3-His (1 and 2 pmols) was added without altering rest of the parameters.

## Additional Information

**How to cite this article**: Anang, S. *et al.* Identification of critical residues in Hepatitis E virus macro domain involved in its interaction with viral methyltransferase and ORF3 proteins. *Sci. Rep.* **6**, 25133; doi: 10.1038/srep25133 (2016).

## Supplementary Material

Supplementary Information

## Figures and Tables

**Table 1:** The X-domain of HEV directly associates with multiple viral proteins: Yeast Two Hybrid analyses of intra-viral interaction partners of the X-domain using *Y2H gold* strain.

Yeast cotransformants	LT^−^	LTHA^−^	LTH^−^A^r+^	LT^−^+ X-α-gal	LTH^−^ + 3-AT (mM)			
					0	5	10	20
AD X + BD ORF3	+++	+++	+++	+++	+++	+++	+++	+++
AD X + BD δ ORF2	+++	−	−	−	−	−	−	−
AD X + BD Met	+++	++	+	+	+++	++	−	−
AD X + BD Y	+++	−	−	−	−	−	−	−
AD X + BD PCP	+++	−	−	−	−	−	−	−
AD X + BD V	+++	−	−	−	−	−	−	−
AD X + BD Helicase	+++	++	−	−	+++	+	−	−
AD X + BD RdRp	+++	++	+	+	+++	+	−	−
AD X + BD	+++	−	−	−	−	−	−	−
AD + BD ORF3	+++	−	−	−	−	−	−	−
AD + BD δ ORF2	+++	−	−	−	−	−	−	−
AD + BD Met	+++	−	−	−	−	−	−	−
AD + BD Y	+++	−	−	−	−	−	−	−
AD + BD PCP	+++	−	−	−	−	−	−	−
AD + BD V	+++	−	−	−	−	−	−	−
AD + BD Helicase	+++	−	−	−	++	−	−	−
AD + BD RdRp	+++	++	+	+	+++	+	−	−
AD + BD	+++	−	−	−	−	−	−	−
AD g3X + BD g3ORF3	+++	+++	+++	+++	+++	+++	+++	+++
AD g3X + BD g3Met	+++	++	+	+	+++	++	−	−
AD TSG101 + BD ORF3	+++	+++	+++	+++	+++	+++	+++	+

*Y2H gold* strain was transformed in indicated combinations and plated on media lacking leucine and tryptophan (LT^−^). Eight random colonies from each cotransformants were replica plated onto media containing various selection markers, as indicated and their growth monitored over a period of four days. g3X, g3ORF3 and g3Met represent corresponding proteins of genotype-3 HEV. AD: Activation domain, BD: Binding domain, +++: Strong growth, ++: Moderate growth, +: Poor growth, −: No growth, L: Leucine, T: Tryptophan, H: Histidine, A: Adenine hemisulfate, A^r^: Aureobasidin, 3-AT: 3-amino 1, 2, 4 Triazole, “−”: Deficiency in the medium, “+”: Supplemented in the medium.

**Table 2:** Methyltransferase inhibits ORF3 binding to the X-domain and vice versa.

Yeast cotransformants	LT^−^ + 1mM M	LTHA^−^+1mM M	LTH^−^A^r+^+1mM M	LT^−^+X-α-gal +1mM M	LTH^−^ + 1mM M + 3-AT (mM)		LTM^−^	LTHAM^−^	LTHM^−^A^r+^	LTM^−^+X-α-gal	LTHM^−^ + 3-AT (mM)	
					5	10					5	10
BD ORF3 Met + AD X	+++	+++	+++	+++	+++	+++	+++	−	−	−	−	−
BD ORF3 + AD X	+++	+++	+++	+++	+++	+++	+++	+++	+++	+++	+++	+++
BD Met ORF3 + AD X	+++	++	+	+	++	−	+++	−	−	−	−	−
BD Met + AD X	+++	++	+	+	++	−	+++	++	+	+	++	−
BD Met ORF3 + AD	+++	−	−	−	−	−	+++	−	−	−	−	−
BD Met + AD	+++	−	−	−	−	−	+++	−	−	−	−	−
BD ORF3 Met + AD	+++	−	−	−	−	−	+++	−	−	−	−	−
BD ORF3 + AD	+++	−	−	−	−	−	+++	−	−	−	−	−
BD Met + AD ORF3	+++	−	−	−	−	−	+++	−	−	−	−	−

*Y2H gold* strain was transformed in indicated combinations and plated on media lacking leucine, tryptophan (LT^−^) and supplemented with 1mM methionine (M). Eight random colonies from each cotransformants were replica plated onto media containing various selection markers, as indicated and their growth monitored over a period of four days. Cotransformants were grown on indicated media in the presence of 1mM methionine (M) or without methionine (M^−^). AD: Activation domain, BD: Binding domain, +++: Strong growth, ++: Moderate growth, +: Poor growth, −: No growth, L: Leucine, T: Tryptophan, H: Histidine, A: Adenine hemisulfate, M: Methionine, A^r^: Aureobasidin, 3-AT: 3-amino 1, 2, 4 Triazole, “−”: Deficiency in the medium, “+”: Supplemented in the medium.

**Table 3:** Analysis of the specificity and selectivity of the interaction between RNA methyltransferases and macro domain proteins.

Yeast cotransformants	LT^−^	LTHA^−^	LTH^−^A^r+^	LT^−^+ X-α-gal	LTH^−^ + 3AT (mM)			
					0	5	10	20
(a) HEV X-protein interaction with human RNA MethylTransferases								
AD X + BD CMTR1	+++	−	−	−	−	−	−	−
AD X + BD CMTR2	+++	−	−	−	−	−	−	−
AD X + BD RG9MTD1	+++	−	−	−	−	−	−	−
AD X + BD RNMT	+++	−	−	−	−	−	−	−
AD + BD CMTR1	+++	−	−	−	−	−	−	−
AD + BD CMTR2	+++	−	−	−	−	−	−	−
AD + BD RG9MTD1	+++	−	−	−	−	−	−	−
AD + BD RNMT	+++	−	−	−	−	−	−	−
(b) HEV Methyltransferase interaction with human macro domains								
BD Met + AD GDAP2	+++	−	−	−	−	−	−	−
BD Met + AD H2FYA2	+++	−	−	−	−	−	−	−
BD Met + AD MacroH2A1.1	+++	−	−	−	−	−	−	−
BD Met + AD C6orf130	+++	−	−	−	−	−	−	−
BD Met + AD PARP14	+++	−	−	−	−	−	−	−
BD Met + AD C20orf133	+++	+++	++	++	+++	−	−	−
BD Met + AD LRP16	+++	+	−	−	+++	−	−	−
BD Met + AD X	+++	++	+	+	+++	++	−	−
BD + AD GDAP2	+++	−	−	−	−	−	−	−
BD + AD H2FYA2	+++	−	−	−	−	−	−	−
BD + AD MacroH2A1.1	+++	−	−	−	−	−	−	−
BD + AD C6orf130	+++	−	−	−	−	−	−	−
BD + AD PARP14	+++	−	−	−	−	−	−	−
BD + AD C20orf133	+++	−	−	−	−	−	−	−
BD + AD LRP16	+++	−	−	−	−	−	−	−
BD + AD X	+++	−	−	−	−	−	−	−
(c) Interaction between human macro domains and RNA MethylTransferases								
BD CMTR1 + AD GDAP2	+++	−	−	−	−	−	−	−
BD CMTR1 + AD H2FYA2	+++	−	−	−	−	−	−	−
BD CMTR1 + AD MacroH2A1.1	+++	−	−	−	−	−	−	−
BD CMTR1 + AD C6orf130	+++	−	−	−	−	−	−	−
BD CMTR1 + AD PARP14	+++	−	−	−	−	−	−	−
BD CMTR1 + AD C20orf133	+++	−	−	−	−	−	−	−
BD CMTR1 + AD LRP16	+++	−	−	−	−	−	−	−
BD CMTR2 + AD GDAP2	+++	−	−	−	−	−	−	−
BD CMTR2 + AD H2FYA2	+++	−	−	−	−	−	−	−
BD CMTR2 + AD MacroH2A1.1	+++	−	−	−	−	−	−	−
BD CMTR2 + AD C6orf130	+++	−	−	−	−	−	−	−
BD CMTR2 + AD PARP14	+++	−	−	−	−	−	−	−
BD CMTR2 + AD C20orf133	+++	++	++	++	+++	−	−	−
BD CMTR2 + AD LRP16	+++	−	−	−	++	−	−	−
BD RG9MTD1 + AD GDAP2	+++	−	−	−	−	−	−	−
BD RG9MTD1 + AD H2FYA2	+++	−	−	−	−	−	−	−
BD RG9MTD1 + AD MacroH2A1.1	+++	−	−	−	−	−	−	−
BD RG9MTD1 + AD C6orf130	+++	−	−	−	−	−	−	−
BD RG9MTD1 + AD PARP14	+++	−	−	−	−	−	−	−
BD RG9MTD1 + AD C20orf133	+++	−	−	−	−	−	−	−
BD RG9MTD1 + AD LRP16	+++	−	−	−	−	−	−	−
BD RNMT + AD GDAP2	+++	−	−	−	−	−	−	−
BD RNMT + AD H2FYA2	+++	−	−	−	−	−	−	−
BD RNMT + AD MacroH2A1.1	+++	−	−	−	−	−	−	−
BD RNMT + AD C6orf130	+++							
BD RNMT + AD PARP14	+++	−	−	−	−	−	−	−
BD RNMT + AD C20orf133	+++	−	−	−	−	−	−	−
BD RNMT + AD LRP16	+++	−	−	−	−	−	−	−

*Y2H gold* strain was transformed in indicated combinations and plated on media lacking leucine, tryptophan (LT^−^). Eight random colonies from each cotransformants were replica plated onto media containing various selection markers, as indicated and their growth monitored over a period of four days. AD: Activation domain, BD: Binding domain, +++: Strong growth, ++: Moderate growth, +: Poor growth, −: No growth, L: Leucine, T: Tryptophan, H: Histidine, A: Adenine hemisulfate, A^r^: Aureobasidin, 3-AT: 3-amino 1, 2, 4 Triazole, “−”: Deficiency in the medium, “+”: Supplemented in the medium.
