# Supplementary material for: Identification of critical residues in Hepatitis E virus macro domain involved in its interaction with viral methyltransferase and ORF3 proteins
Source: Sci Rep. 2016 Apr 26;6:25133. doi: 10.1038/srep25133 (PMC4844956; doi:10.1038/srep25133)
Supplement: Supplementary Information [file srep25133-s1.pdf]

Identification of critical residues in Hepatitis E virus macro domain involved in its interaction with viral methyltransferase and ORF3 proteins.

Saumya Anang[1], Chandru Subramani[1,4], Vidya P. Nair[1,4], Sheetal Kaul[1], Nidhi Kaushik[1], Chandresh Sharma[2], Ashutosh Tiwari[2,3], CT Ranjith-Kumar [1], Milan Surjit[1*]

[1]Virology Laboratory, Vaccine and Infectious Disease Research Centre, Translational Health Science and Technology Institute, NCR Biotech Science Cluster, 3rd Milestone, Faridabad-Gurgaon Expressway, P O Box No. 04, Faridabad-121001, Haryana, India.

[2]Centre for Bio-design & Diagnostics, Translational Health Science and Technology Institute, NCR Biotech Science Cluster, 3rd Milestone, Faridabad-Gurgaon Expressway, P O Box No. 04, Faridabad-121001, Haryana, India.

[3]Experimental Medicine and Biotechnology Department, Postgraduate Institute of Medical Education and Research (PGIMER), Chandigarh, India.

[4] These authors contributed equally to this work.

[*] Correspondence and requests for materials should be addressed to MS; milan@thsti.res.in

Supplementary Fig. S1

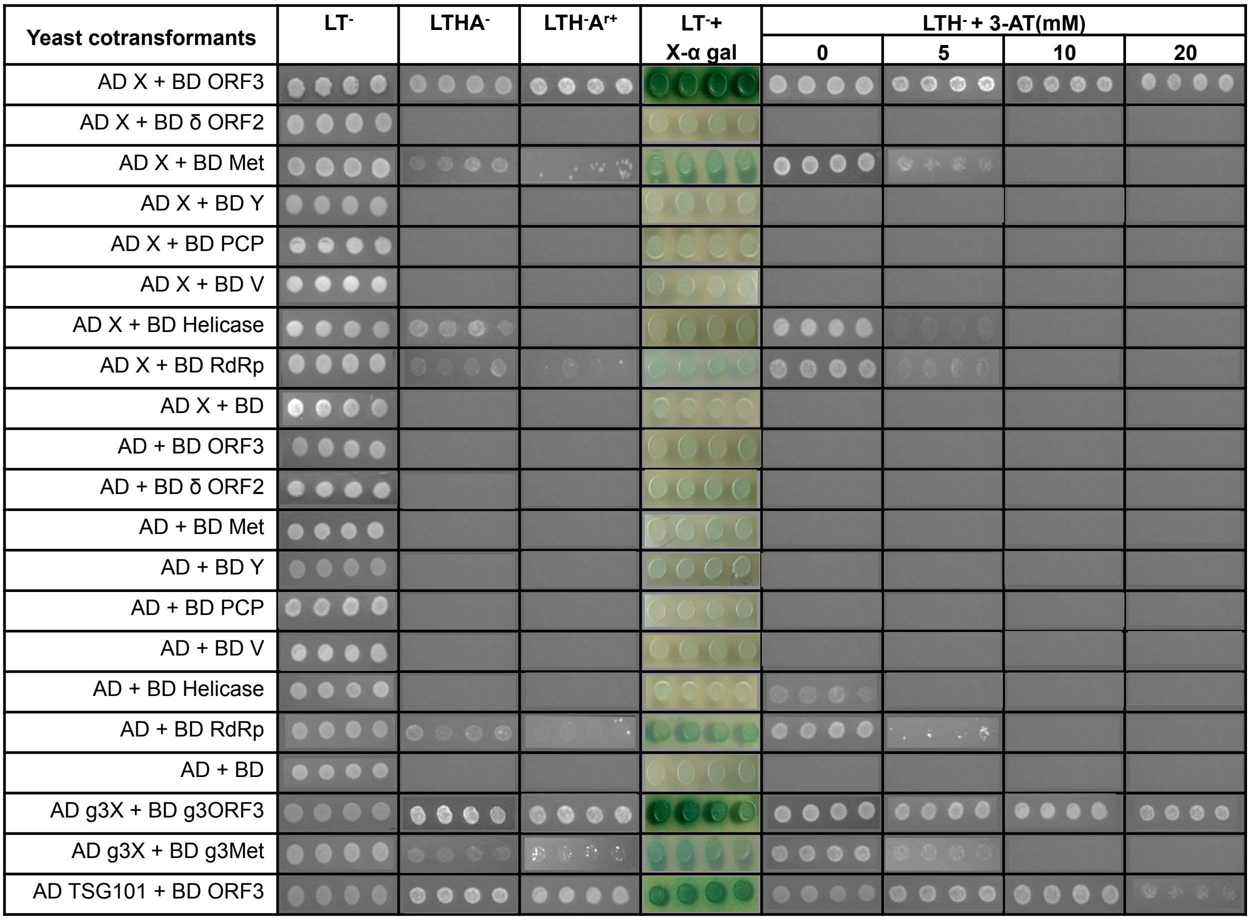

Supplementary Fig. S2

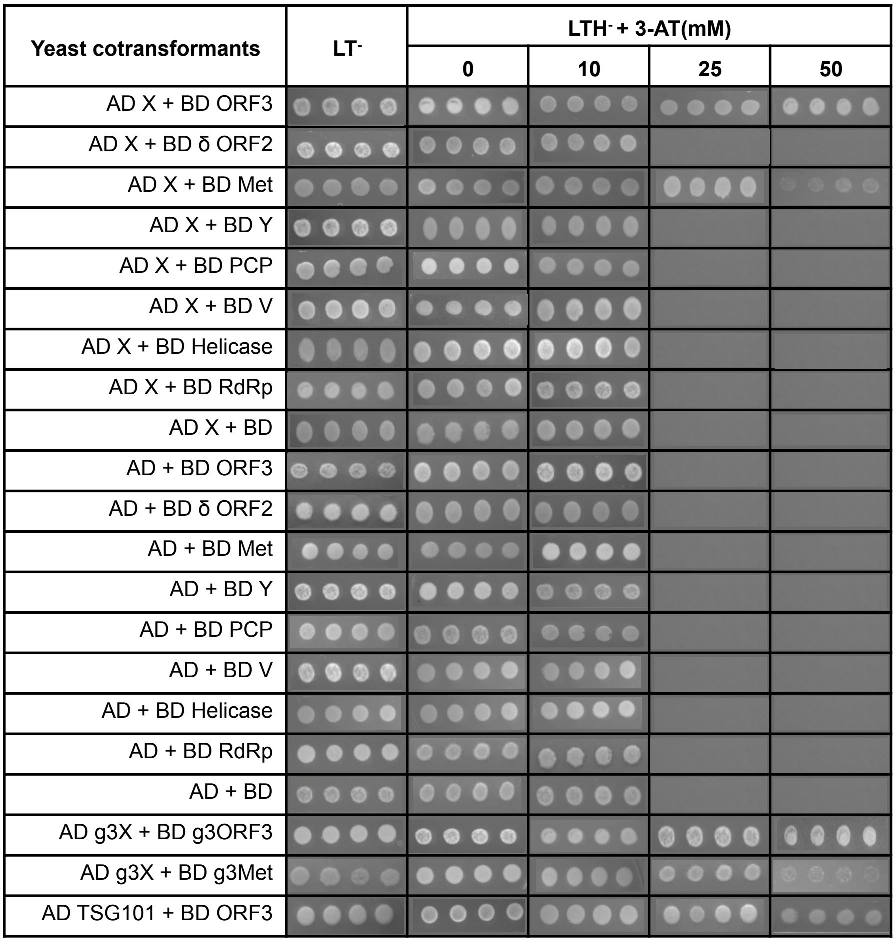

Supplementary Fig. S3

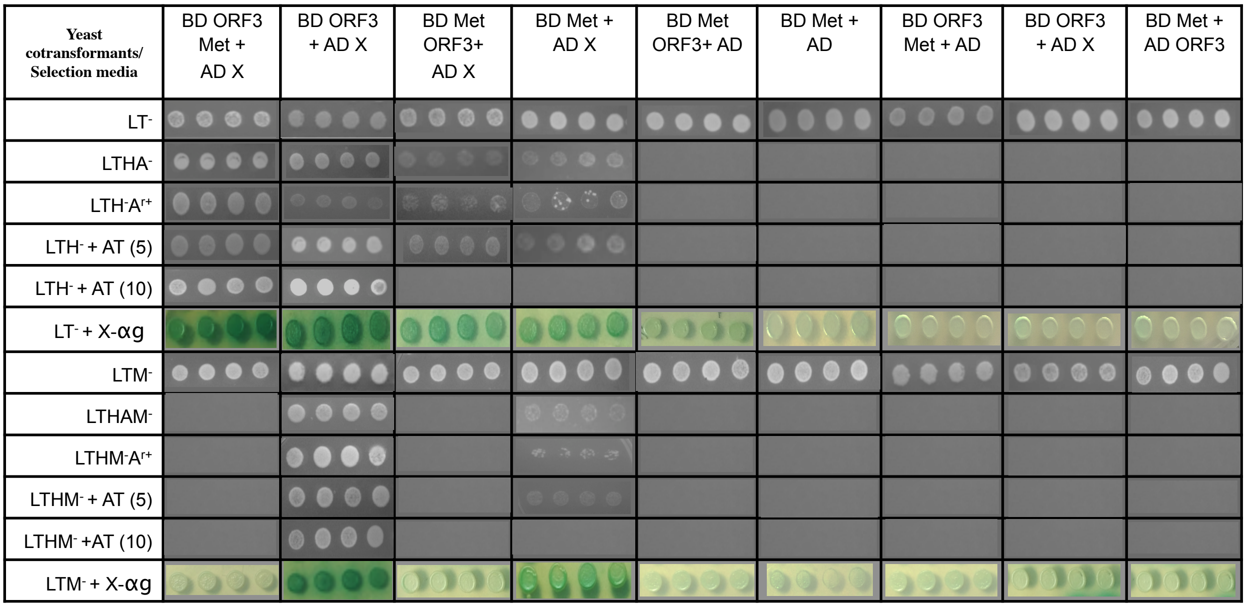

Legend to Supplementary Figures

Supplementary Fig. S1. Y2H analysis of intra-viral interaction partners of the HEV X-domain in *Y2H Gold* strain.

Illustration of colonies that represent the data of Table 1.

Supplementary Fig. S2. Y2H analysis of intra-viral interaction partners of the HEV X-domain in *Mav 203* strain.

Illustration of colonies that represent the data of supplementary Table S1.

Supplementary Fig. S3. Y3H analysis of the interaction between the HEV X-domain, methyltransferase and ORF3 in *Y2H Gold* strain.

Illustration of colonies that represent the data of Table 3.

Supplementary Table S1. Yeast Two Hybrid analyses of intra-viral interaction partners of the X-domain using *Mav203* strain.

Yeast cotransformants	LT^-	LTH^- + 3-AT(mM)			
		0	10	25	50
AD X + BD ORF3	+++	+++	+++	+++	+++
AD X + BD δ ORF2	+++	+++	+++	-	-
AD X + BD Met	+++	+++	+++	+++	++
AD X + BD Y	+++	+++	+++	-	-
AD X + BD PCP	+++	+++	+++	-	-
AD X + BD V	+++	+++	+++	-	-
AD X + BD Helicase	+++	+++	+++	-	-
AD X + BD RdRp	+++	+++	+++	-	-
AD X + BD	+++	+++	+++	-	-
AD + BD ORF3	+++	+++	+++	-	-
AD + BD δ ORF2	+++	+++	+++	-	-
AD + BD Met	+++	+++	+++	-	-
AD + BD Y	+++	+++	+++	-	-
AD + BD PCP	+++	+++	+++	-	-
AD + BD V	+++	+++	+++	-	-
AD + BD Helicase	+++	+++	+++	-	-
AD + BD RdRp	+++	+++	+++	-	-
AD + BD	+++	+++	+++	-	-
AD g3X + BD g3ORF3	+++	+++	+++	+++	+++
AD g3X + BD g3Met	+++	+++	+++	+++	++
AD TSG101 + BD ORF3	+++	+++	+++	+++	+++

Mav203 strain was transformed in indicated combinations and plated on media lacking leucine, tryptophan (LT^-). Eight random colonies from each cotransformants were replica plated onto media containing various selection markers, as indicated and their growth monitored over a period of four days. AD: Activation domain, BD: Binding domain, +++: Strong growth, ++: Moderate growth, +: Poor growth, -: No growth, L: Leucine, T: Tryptophan, H: Histidine, 3-AT: 3-amino 1, 2, 4 Triazole, “-”: Deficiency in the medium, “+”: Supplemented in the medium.

Supplementary Table S2 : Detailed procedure for generating the constructs used in this study.

Clone Name	Cloning Strategy
pBRIDGE ORF3	ORF3 sequence was PCR amplified with Fp ORF3 and Rp ORF3 primers using pSKHEV2 as template. The amplified product was digested with EcoRI and SalI and ligated with EcoRI and SalI digested pBRIDGE vector.
pGBKT7 ORF3	ORF3 sequence was PCR amplified with Fp ORF3 and Rp ORF3 primers using pSKHEV2 as template. The amplified product was digested with EcoRI and SalI and ligated with EcoRI and SalI digested pGBKT7 vector.
pGADT7 ORF3	As described[22].
pGBKT7Δ (112-608) ORF2	pPiczA ΔORF2 (112-608) was digested with NotI and XbaI. pGBKT7 digested with NcoI and blunted. Both fragments were then ligated.
pGADT7 Met, pGADT7 Y, pGADT7 PCP, pGADT7 V,pGADT7 RdRp, pGADT7 X	Methyltransferase, Y, PCP,V, RdRp and X sequences were PCR amplified with Fp and Rp Met, Fp and Rp Y, Fp and Rp PCP, Fp and Rp V, Fp and Rp RdRp, Fp and Rp X primers using pSKHEV2 as template. The amplified products were digested with EcoRI and XhoI and ligated with pGADT7 vector digested with EcoRI and XhoI.
pGADT7 Helicase	The helicase sequence was PCR amplified with Fp and Rp Helicase primers using pSKHEV2 as template. The amplified product was digested with NdeI and EcoRI and ligated with pGADT7 vector digested with NdeI and EcoRI.
pGBKT7 Met, pGBKT7 Y, pGBKT7 PCP, pGBKT7 V, pGBKT7 RdRp, pGBKT7 X	All these clones were generated by sub cloning. pGADT7 Met, pGADT7 Y, pGADT7 PCP, pGADT7 V, pGADT7 RdRp and pGADT7 X were digested with EcoRI and XhoI. 931, 682, 483, 202, 1462 and 475 base pair fragments were gel extracted respectively and ligated with pGBKT7 vector digested with EcoRI and SalI.
pBRIDGE Met	The clone was generated by digesting pGADT7 Met with EcoRI and XhoI. 931 base pair fragment was gel extracted and ligated with EcoRI and SalI digested pBRIDGE vector.
pGBKT7 Helicase	The clone was generated by digesting pGADT7 Helicase with NdeI and EcoRI. Gel extracted 783 base pair was ligated with pGBKT7 vector digested with NdeI and EcoRI.
pGADT7 X (1-124)	pGADT7 X was digested with Sma1 and XhoI. 8359 base pair fragment was gel extracted, end filled and self -ligated.
pGBKT7 D1-ORF3	1-35 amino acids of ORF3 was PCR amplified with Fp ORF3 and Rp ORF3 1-35 primers using pSKHEV2 as template. The amplified product was digested with EcoRI and SalI and ligated with EcoRI and SalI digested pGBKT7 vector.
pGBKT7 D2-ORF3, pGBKT7 D3-ORF3, pGBKT7 D4-ORF3, pGBKT7 D6-ORF3	As described[38].
pGBKT7 D5-ORF3	25-68 amino acids of ORF3 was PCR amplified with Fp and Rp ORF3 25-68 primers using pSKHEV2 as template. The amplified product was digested with EcoRI and SalI and ligated with EcoRI and SalI digested pGBKT7 vector.
pGBKT7 D7-ORF3	63-123 amino acids of ORF3 was PCR amplified with Fp ORF3 63-123 and Rp ORF3 primers using pSKHEV2 as template. The amplified product was digested with EcoRI and SalI and ligated with EcoRI and SalI digested pGBKT7 vector.

pGADT7 D1-Met	1-30 amino acids of methyltransferase was PCR amplified with Fp Met and Rp Met 1-30 primers using pSKHEV2 as template. The amplified product was digested with EcoRI and XhoI and ligated with EcoRI and XhoI digested pGADT7 vector.
pGADT7 D2-Met	1-60 amino acids of methyltransferase was PCR amplified with Fp Met and Rp Met 1-60 primers using pSKHEV2 as template. The amplified product was digested with EcoRI and XhoI and ligated with EcoRI and XhoI digested pGADT7 vector.
pGADT7 D3-Met	1-90 amino acids of methyltransferase was PCR amplified with Fp Met and Rp Met 1-90 primers using pSKHEV2 as template. The amplified product was digested with EcoRI and XhoI and ligated with EcoRI and XhoI digested pGADT7 vector.
pGADT7 D4-Met	pGADT7 Met was digested with SmaI and XhoI. 8743 base pair was gel extracted and self -ligated.
pGADT7 D5-Met	40-90 amino acids of methyltransferase was PCR amplified with Fp Met 40-90 and Rp Met 1-90 primers using pSKHEV2 as template. The amplified product was digested with EcoRI and XhoI and ligated with EcoRI and XhoI digested pGADT7 vector.
pGADT7 D6-Met	pGADT7 Met was digested with NcoI. After end filing, it was digested with XhoI and 489 base pair was gel extracted. pGADT7 vector was digested with BamHI, end filled and then digested with XhoI. The gel extracted fragment was ligated with pGADT7 vector digested with BamHI and XhoI.
pGADT7 D7-Met	pGADT7 Met was digested with SmaI and XhoI. 8137 base pair was gel extracted and self -ligated.
pGADT7 D1-X	pGADT7 X was digested with NotI. After end filling, it was digested with XhoI. The 8155 base pair fragment containing 1-56 amino acids coding region of X protein was gel extracted and ligated.
pGADT7 D2-X	5-32 amino acids of X was PCR amplified with Fp and Rp KGG primers using pSKHEV2 as template. The amplified product was digested with EcoRI and XhoI and ligated with EcoRI and XhoI digested pGADT7 vector.
pGADT7 D3-X	25-64 amino acids of X was PCR amplified with Fp and Rp VDHTPR primers using pSKHEV2 as template. The amplified product was digested with EcoRI and XhoI and ligated with EcoRI and XhoI digested pGADT7 vector.
pGADT7 D4-X	47-86 amino acids of X was PCR amplified with Fp and Rp AASEAA primers using pSKHEV2 as template. The amplified product was digested with EcoRI and XhoI and ligated with EcoRI and XhoI digested pGADT7 vector.
pGADT7 D5-X	47-118 amino acids of X was PCR amplified with Fp AASEAA and Rp APDDAW primers using pSKHEV2 as template. The amplified product was digested with EcoRI and XhoI and ligated with EcoRI and XhoI digested pGADT7 vector.
pGADT7 D6-X	pGADT7 X was digested with NotI. After end filling, it was digested with EcoRI. The 8293 base pair fragment containing 56-158 amino acids of X protein was gel extracted and ligated. Clone was named as pGADT7 X (56-158) which was then digested with KpnI. After blunting, it was digested with EcoRI. 120 base pair fragment was gel extracted and ligated with pGADT7 between EcoRI and SmaI sites.
pGADT7 D7-X	70-118 amino acids of X was PCR amplified with Fp and Rp APDDAW primers using pSKHEV2 as template. The amplified product was digested with EcoRI and XhoI and ligated with EcoRI and XhoI digested pGADT7 vector.

pGADT7 D8 II-EE X	PCR was done with Fp pBridgeX II-EEmutNotI and Rp APDDAW primers using pSKHEV2 as template and digested with NotI and XhoI. pGADT7 X was digested with NotI and XhoI. 5565 and 2488bp fragment was gel extracted and ligated with the PCR product.
pGADT7 D9 R-E X	pGADT7 X digested with KpnI. 7632 and 565bp fragments were gel extracted. 164 base pair PCR amplified with Fp pBridgeX L-RmutKpnI and Rp pBridgeX R-EmutKpnI primers using pSKHEV2 as template and digested with KpnI. All the three fragments were ligated.
pBRIDGE D10 L-R X	PCR was done with Fp pBridge X L-RmutKpnI and Rp pBridgeX L-RmutKpnI primers using pSKHEV2 as template and digested with KpnI. pBRIDGE X was digested with KpnI. 6811 base pair fragment was gel extracted and ligated with KpnI digested PCR product.
pBRIDGE D11 LL-EE X	PCR was done with Fp pBridgeX LL-EEmutKpnI and Rp-APDDAW primers using pSKHEV2 as template and digested with KpnI. pGADT7 X was digested with KpnI and 164bp fragment was gel extracted. pBRIDGE X was digested with PstI, blunted and then digested with KpnI. 6627 fragment was gel extracted. All the three fragments were ligated.
pBRIDGE D12 R-E and LL-EE X	PCR was done with Fp pBridgeX LL-EEmutKpnI and Rp APDDAW primers using pSKHEV2 as template and digested with KpnI. pGADT7 D9 R-E X was digested with KpnI and 164bp fragment was gel extracted. pBRIDGE X was digested with PstI, blunted and then digested with KpnI. 6627 fragment was gel extracted. All the three fragments were ligated.
pAS2 ORF2	As described[16].
pBRIDGE ORF3 Met	Methyltransferase sequence was PCR amplified with Fp and Rp Metpbridgemcs2 primers using pSKHEV2 as template. The amplified product was digested with NotI and BglII and ligated with NotI and BglII digested pBRIDGE ORF3.
pBRIDGE Met ORF3	ORF3 sequence was PCR amplified with Fp and Rp ORF3pbridgemcs2 primers using pSKHEV2 as template. The amplified product was digested with NotI and BglII and ligated with NotI and BglII digested pBRIDGE Met.
pGEX4T1 X	X encoding region was PCR amplified with Fp X and Rp APDDAW primers using pSKHEV2 as template. The amplified product was digested with EcoRI and XhoI and ligated with pGEX4T1 vector digested with EcoRI and XhoI.
pGEX4T1 Mut X	pBRIDGE D11 LL-EE X was digested with EcoRI and XhoI, 354 base pair was gel extracted and ligated with EcoRI and XhoI digested pGEX4T1 vector.
pET28a Met	The clone was generated by digesting pGADT7 Met with EcoRI and XhoI. 931 base pair fragment was gel extracted and ligated with pET-28a vector digested with EcoRI and XhoI.
pRSET ORF3	As described[47].
pGBKT7 hCMTR1	hCMTR1 cloned in pGADT7 vector between NdeI and XhoI was digested with the same. 2500bp fragment was gel extracted and ligated with NdeI and SalI digested pGBKT7 vector.
pGBKT7 hCMTR2	hCMTR2 cloned in pGADT7 vector between NdeI and XhoI was digested with the same. 2300bp fragment was gel extracted and ligated with NdeI and SalI digested pGBKT7 vector.

pGBKT7 hRG9MTD1	hRG9MTD1 cloned in pGADT7 vector between EcoRI and XhoI was digested with the same. 1200bp fragment was gel extracted and ligated with EcoRI and SalI digested pGBKT7 vector.
pGBKT7 hRNMT	hRNMT cloned in pGADT7 vector between NdeI and XhoI was digested with the same. 1400bp fragment was gel extracted and ligated with NdeI and SalI digested pGBKT7 vector.
pGADT7 hGDAP2	hGDAP2 sequence was PCR amplied using cDNA from A549 cells. The amplified product was digested with EcoRI and XhoI and ligated with EcoRI and XhoI digested pGADT7 vector.
pGADT7 hH2FYA2	hH2FYA2 sequence was PCR amplied using cDNA from Huh7 cells. The amplified product was digested with NdeI and SmaI and ligated with NdeI and SmaI digested pGADT7 vector.
pGADT7 hMacroH2A1.1	hMacroH2A1.1 sequence was PCR amplied using cDNA from A549 cells. The amplified product was digested with EcoRI and XhoI and ligated with EcoRI and XhoI digested pGADT7 vector.
pGADT7 hC6orf130	hC6orf130 sequence was PCR amplied using cDNA from Huh7 cells. The amplified product was digested with EcoRI and XhoI and ligated with EcoRI and XhoI digested pGADT7 vector.
pGADT7 hPARP14	hPARP14 sequence was PCR amplied using cDNA from Huh7 cells. The amplified product was digested with EcoRI and XhoI and ligated with EcoRI and XhoI digested pGADT7 vector
pGADT7 hC20orf133	hC20orf133 sequence was PCR amplied using cDNA from A549 cells. The amplified product was digested with EcoRI and XhoI and ligated with EcoRI and XhoI digested pGADT7 vector.
pGADT7 hLRP16	hLRP16 sequence was PCR amplied using cDNA from A549 cells. The amplified product was digested with NdeI and EcoRI and ligated with NdeI and EcoRI digested pGADT7 vector.
pUNO X-HA	X sequence was PCR amplified with Fp pCDNA5 X and Rp X geno3 HA primers using pSKHEV2 as template. The amplified product was digested with NheI and BamHI and ligated with NheI and BamHI digested pUNO MCS2 vector.
pUNO X Mut-HA	X sequence was PCR amplified with Fp pCDNA5 X and Rp X geno3 HA primers using pBRIDGE D11 LL-EE X as template. The amplified product was digested with NheI and BamHI and ligated with NheI and BamHI digested pUNO MCS2 vector.
pCDNA5 Met-Flag	Methyltransferase sequence was PCR amplified with Fp and Rp pCDNA5 Met Flag primers using pSKHEV2 as template. The amplified product was digested with HindIII and ligated with HindIII and EcoRV digested pCDNA5 vector.
pVITRO2 ORF3-myc	The ORF3 sequence was PCR amplified with Fp Gal4 BD Sequencing primer and RpORF3 using pGBKT7 ORF3 as template. The amplified product was digested with SalI and ligated with EcorRV and SalI digested pVITRO2 vector.
pUNO helicase myc	Helicase sequence with C-terminal myc tag was PCR amplified using Fp and Rp pUNO helicase myc primers, digested with AgeI, NheI and ligated into pUNO vector digested with same.
pGADT7 X geno 3	The X genotype3 sequence was PCR amplified with Fp and Rp Xgeno3 primers. The amplified product was digested with NdeI and BamHI and ligated with NdeI and BamHI digested pGADT7 vector.

pGBKT7 Met geno3	Methyltransferase genotype3 sequence was PCR amplified with Fp Rp Met geno3 primers, digested with EcoRI and SalI and ligated with EcoRI and SalI digested pGBKT7 vector.
pGBKT7 ORF3 geno3	pUNO ORF3 geno3 DNA was digested with AgeI and NheI and end filled. ORF3 band was gel extracted and ligated with pGBKT7 vector that was digested with NcoI and end filled.

Supplementary Table S3 : List of primers used in the study.	
**PRIMER NAME**	**SEQUENCE**
Fp ORF3	5' TCGCCGGAATTCGGTTCGCGACCATGCGCCC 3'
Rp ORF3	5' AGACTAGTCGACGGCGGCGCGGCCCCAGCT 3'
Fp Met	5' TGCTCAGAATTCGTTAGGCCTTTTCTCTCTC 3'
Rp Met	5' AGACTGCTCGAGTTAGTAAGTAAGTCATTAGGCGGG 3'
Fp Y	5' TGCTCAGAATTCCGCGTTGTGGTGACGTATG 3'
Rp Y	5' AGACTGCTCGAGTTAAAAGCCGGCCGAGAGC 3'
Fp PCP	5' TGCTCAGAATTCGCTCAGTGTAGGCGCTG 3'
Rp PCP	5' AGACTGCTCGAGTTAGAGATTGTGGCGCTCTGG 3'
Fp V	5' TGCTCAGAATTCCCTAGTCCAGCCCAGCCC 3'
Rp V	5' AGACTGCTCGAGTTAGCGATGCCGGGCCGT 3'
Fp RdRp	5' TGCTCAGAATTCGGTGGCGAAATTGGC 3'
Rp RdRp	5' AGACTGCTCGAGTTATTCCACCCGACACAGA 3'
Fp X	5' TGCTCAGAATTCCCGGATGGCTCTAAGGTG 3'
Rp X	5' AGACTGCTCGAGTTATGCTGTCCGCGCAAC 3'
Fp Helicase	5' TGCTCACATATGGGCTGTCGAGTC 3'
Rp Helicase	5' AGACTGGAATTCTTAGAAAAAGTTATTAACGAT 3'
Rp ORF3 1-35	5' AGACTAGTCGACGTGGCGCGGGCAGCATA 3'
Fp ORF3 25-68	5' ACGTATGGAATTCTGCCTATGCTGCCCGC 3'
Rp ORF3 25-68	5' AGACTGGTCGACTTACGAAGGGCTGAGAATCAAC 3'
Fp ORF3 63-123	5' TCGCCGGAATTCAGCCCTTCGCAATCCCCTAT 3'
Rp Met 1-30	5' AGACTGCTCGAGTTACCAGAAAACCTCGGGG 3'
Rp Met 1-60	5' AGACTGCTCGAGTTAGGGGTGGGCACCA 3'
Rp Met 1-90	5' AGACTGCTCGAGTTAGGTAGGGGCAGTATACC 3'
Fp Met 40-90	5' TGCTCAGAATTC AATGAGCTGGAGCTTTAC 3'
Fp KGG	5' TCGCCGGAATTCAAGGTGTTCGCCGGCTCGCTGTTTGA 3'
Rp KGG	5' TCGCCGCTCGAGCCCACCGCCAGGGCGGTGGT 3'
Fp VDHTPR	5' TCGCCGGAATTCGTTGACCACCGCCCT 3'
Rp VDHTPR	5' TCGCCGCTCGAGCCGGGGGGTTAATGTGT 3'
Fp AASEAA	5' TCGCCGGAATTCGCTGCCTCTTTTGTG 3'
Rp AASEAA	5' TCGCCGCTCGAGGGCAGCCTCAAGC 3'
Fp APDDAW	5' TCGCCGGAATTCGCTCCTGATTATAGGT 3'
Rp APDDAW	5' TCGCCGCTCGAGCCAGGCGTCAAAA 3'
Fp pBridgeX II-EEmutNot1	5' GACGGCGCGGCCGCCTACACATTAACCCCCCGGCCAGA GGAACATGCCGTCGCTCCTGAT 3'
Rp pBridgeX R-EmutKpn1	5' GCAGCGGTACCGAGTTCGGAGCAAGTCT 3'
Fp pBridge X L-RmutKpn1	5' ACCAGAGGTACCCCGCCT 3'
Rp pBridgeX L-RmutKpn1	5' TGCAGCGGTACCGCGGCGGGAGCAAG 3'

Fp pBridgeX LL-EEmutKpn1	5' CGCCTCGGTACCGCTGCATACCCAGAAGAAGGGACCGG CATATAC 3'
Fp Metpbridgemcs2	5' GAACTAGCGGCCGCAGTTAGGCCTTTTCTCTCTC 3'
Rp Metpbridgemcs2	5' AGATAGATCTTTAGTAAGTCATTAGGCGGG 3'
Fp ORF3pbridgemcs2	5' GAACTAGCGGCCGCAGGTTCGCGACCATGCGCCC 3'
Rp ORF3pbridgemcs2	5' AGATAGATCTTTAGGCGGCGCGGCCCCAGCT 3'
Fp hCMTR1	5' ATCTACATATGGCCATGAAGAGGAGAACTGACCCAG 3'
Rp hCMTR1	5' TCTACCTCGAGGGCCCTGTGCATCTGG 3'
Fp hCMTR2	5' ATCTACATATGGCCATGAGTAAGTGCAGAAAGACACCA 3'
Rp hCMTR2	5' TCTACCTCGAGGTTTTGTAACTGAAGGCTGTTGA 3'
Fp hRG9MTD1	5' ATCTAGAATTCGCCATGAGTGTTAGTGTCAATTTCTTCAGA 3
Rp hRG9MTD1	5' TCTACCTCGAGAGTCTTTGCCTTCTTTAGTCTGTTG 3'
Fp hRNMT	5' ATCTACATATGGCCATGGCAAATTCTGCAAAAGC 3'
Rp hRNMT	5' TCTACCTCGAGCTGCTGTTTCTCAAAGGCAA 3'
Fp hGDAP2	5' ATCTAGAATTCGCCATGGATCCCTTAGGTGCACC 3'
Rp hGDAP2	5' TCTACCTCGAGCAAATCTGGTGATGGGGGAT 3'
Fp hMACROH2A1.1	5' ATCTAGAATTCGCCATGTCGAGCCGCGGT 3'
Rp hMACROH2A1.1	5' TCTACCTCGAGGTTGGCGTCCAGCTTGG 3'
Fp hC6orf133	5' ATCTAGAATTCGCCATGGCCAGCAGCCTTAAT 3'
Rp hC6orf133	5' TCTACCTCGAGGAGTGTGTACACAGTAATTTTGATGTC 3'
Fp hPARp14	5' ATCTAGAATTCGCCATGGTGCTGATTGTGCAGC 3'
Rp hPARp14	5' TCTACCTCGAGTATGGCTTCAGCAACCTTATCTG 3'
Fp hC20orf133	5' ATCTAGAATTCGCCATGTACCCCAGCAACAAGAAG 3'
Rp hC20orf133	5' TCTACCTCGAGTTTAGTTCCATTTCTTTGTTCCTTC 3'
Fp hLRp16	5' ATCTACATATGGCCATGTCTCTACAGAGCCGACTGTC 3'
Rp hLRp16	5' TCTACGAATTCGGCCACGGGGAAGTAGTG 3'
Fp PCDNA5 X	5' ATACTAGGATCCGACATGGCGCCGGATGGCTCTAAGGTG 3'
Rp X geno3 HA	5' GACGCTAGCTCAAGCGTAATCTGGAACATCGTATGGGTA GGCCGTACGAGCCGTATCCTCAGTTATGG 3'
Fp pCDNA5 Met Flag	5' ATACAAGCTTGACATGGTTAGGCCTTTTCTCTCTC 3'
Rp pCDNA5 Met Flag	5' CTTACTTGTCATCGTCGTCCTTGTAGTCGTAAGTCATTAG GCGGG 3'
Fp pUNO helicase Myc	5' GACACCGGTCATCATGGGCTGTCGAGTCACCCCCG 3'
Rp pUNO helicase Myc	5' GACGCTAGCTCACAGGTCTTCTTCAGAGATCAGTTTCTGT TCGAAAAAGTTATTAACGATTGCATCGGAG 3'
Fp Gal4 BD Sequencing primer	5' TCATCGGAAGAGAGTAGTAACAAAGGTCAAAGACAGTTG ACTGTATCG 3'
Fp X geno3	5' ACGCTCATATGGTGTATGCAGGGTCATTGTTTG 3'
Rp X geno3	5' ATGCGGATCCACCCAGGCATCAAAACTGAG 3'
Fp Met geno3	5' TCGCCGGAATTCGAGGCCCACCAGTTCATTAAG 3'
Rp Met geno3	5' AGACTAGTCGACGATCCACGCACGAAGTATGG 3'
